# Efficacy of mRNA-1273 and Novavax ancestral or BA.1 spike booster vaccines against SARS-CoV-2 BA.5 infection in non-human primates

**DOI:** 10.1126/sciimmunol.adg7015

**Published:** 2023-05-16

**Authors:** Nanda Kishore Routhu, Samuel David Stampfer, Lilin Lai, Akil Akhtar, Xin Tong, Dansu Yuan, Taras M. Chicz, Ryan P. McNamara, Kishor Jakkala, Meredith E. Davis-Gardner, E. Lovisa St Pierre, Brandon Smith, Kristyn Moore Green, Nadia Golden, Breanna Picou, Sherrie M. Jean, Jennifer Wood, Joyce Cohen, Ian N. Moore, Nita Patel, Mimi Guebre-Xabier, Gale Smith, Greg Glenn, Pamela A. Kozlowski, Galit Alter, Rafi Ahmed, Mehul S. Suthar, Rama Rao Amara

**Affiliations:** ^1^ Division of Microbiology and Immunology, Emory Vaccine Center, Emory National Primate Research Center, Emory University, Atlanta, GA 30329, USA.; ^2^ Department of Microbiology and Immunology, Emory School of Medicine, Emory University, Atlanta, GA 30322, USA.; ^3^ Division of Infectious Diseases, Department of Medicine, Emory University School of Medicine, Atlanta, GA, USA.; ^4^ Emory Vaccine Center, Division of Microbiology and Immunology, Emory National Primate Research Center, Emory University, Atlanta, GA 30329, USA; Department of Pediatrics, Division of Infectious Diseases, Emory University School of Medicine, Atlanta, GA 30322, USA.; ^5^ Emory Vaccine Center, Department of Microbiology and Immunology, Emory University School of Medicine, Atlanta, GA.; ^6^ Ragon Institute of MGH, MIT and Harvard, Cambridge, MA 02139, USA.; ^7^ Tulane National Primate Research Center, Covington, LA, USA.; ^8^ Division of Animal Resources, Emory National Primate Research Center, Emory University, Atlanta, GA 30329, USA.; ^9^ Department of Psychiatry and Behavioral Sciences, Emory School of Medicine, Emory University, Atlanta, GA 30322, USA.; ^10^ Division of Pathology, Emory National Primate Research Center, Emory University, Atlanta, GA 30329, USA.; ^11^ Novavax, Inc., 21 Firstfield Road, Gaithersburg, MD 20878, USA.; ^12^ Department of Microbiology, Immunology, and Parasitology, Louisiana State University Health Sciences Center, New Orleans, Louisiana 70112, USA.; ^13^ Department of Pediatrics, Division of Infectious Diseases Vaccine Center, Emory Vaccine Center, Yerkes National Primate Research Center, Emory University School of Medicine, Atlanta, GA 30329.

## Abstract

Omicron SARS-CoV-2 variants escape vaccine-induced neutralizing antibodies and cause nearly all current COVID-19 cases. Here, we compared the efficacy of three booster vaccines against Omicron BA.5 challenge in rhesus macaques: mRNA-1273, the Novavax ancestral spike protein vaccine (NVX-CoV2373), or Omicron BA.1 spike protein version (NVX-CoV2515). All three booster vaccines induced a strong BA.1 cross-reactive binding antibody and changed immunoglobulin G dominance from IgG1 to IgG4 in the serum. All three booster vaccines also induced strong and comparable neutralizing antibody responses against multiple variants of concern, including BA.5 and BQ.1.1, along with long-lived plasma cells in the bone marrow. The ratio of BA.1 to WA-1 spike-specific antibody-secreting cells in the blood was higher in NVX-CoV2515 animals compared to NVX-CoV2373 animals, suggesting a better recall of BA.1 specific memory B cells by the BA.1 spike-specific vaccine compared to the ancestral spike-specific vaccine. Further, all three booster vaccines induced low levels of spike-specific CD4 but not CD8 T cell responses in the blood. Following challenge with SARS-CoV-2 BA.5 variant, all three vaccines showed strong protection in the lungs and controlled virus replication in the nasopharynx. In addition, both Novavax vaccines blunted viral replication in nasopharynx at day 2. The protection against SARS-CoV-2 BA.5 infection in the upper respiratory airways correlated with binding, neutralizing, and ADNP activities of the serum antibody. These data have important implications for COVID-19 vaccine development, as vaccines that lower nasopharyngeal virus may help to reduce transmission.

## Teaser

Boosting with mRNA-1273 or NVX-CoV2373 vaccine enhances neutralizing antibody response and protection against SARS-COV-2 BA.5 in NHPs.

## INTRODUCTION

Severe acute respiratory syndrome coronavirus 2 (SARS-CoV-2) has caused millions of infections and deaths since 2019, with ongoing worldwide circulation still happening today (*1*, *2*). Its continued evolution has resulted in the emergence of a number of variants of concern (VOC), which possess enhanced resistance to the immunity induced by current COVID-19 vaccines as well as greater replication fitness and transmissibility (*3*, *4*). In particular, the B.1.1.529 (Omicron BA.1) VOC isolate contains 37 mutations in the spike protein, 15 of which are in the Receptor Binding Domain (RBD) (*5*). Such mutations resulted in its rapid dominance, displacing prior variants to become responsible for >99% of infections in the United States in mid-January 2022 (*1*, *2*). Its sub-lineages of BA.2, BA.5, BA.2.75, and (more recently) BQ1.1 have even greater immune evasion and transmissibility (*6*), successively replacing the current Omicron variant to become dominant variants globally (*1*, *2*). Therefore, developing an effective COVID-19 vaccine that can restrict the emergence of SARS-CoV-2 VOCs and their transmissions remained a public health priority.

The waning of vaccine-induced immune responses is well reported in vaccinated individuals, with or without prior SARS-CoV-2 infection (*7*, *8*), and is a major concern for the current COVID-19 vaccines. Breakthrough infections after vaccination help drive ongoing SARS-CoV-2 transmission and the emergence of new VOCs (*9*). Current FDA-approved COVID-19 vaccines provide incomplete protection against mild COVID-19 and transmission even shortly after booster vaccination, with waning protection against severe COVID-19 in the long-term (*1*, *2*). Compared to the original primary series vaccination, booster doses provide a superior immune response against VOCs, including BA.1 Omicron and its sub-lineages (*10*), but these responses wane quickly (*11*–*14*). With the multiple vaccines and immunization strategies available, along with waning antibody titers in circulation and mucosal compartments (*1*, *2*), it is critical to determine the best vaccine regimens to maximize durable immune responses against emerging VOCs. One strategy is to use a booster with a spike protein sequence matching circulating VOCs, but this has provided no advantage compared to boosting with the original wild-type sequence vaccine (*15*–*17*).

Durable mucosal immunity is a key component in protecting against SARS-CoV-2 and emerging VOCs as they infect the respiratory airways, primarily at the upper and lower respiratory mucosal surfaces (*18*, *19*). Heterologous subunit-based adjuvanted vaccines are known to induce robust and durable immune responses in the circulation and high IgA levels in the mucosae if given by a mucosal route (*19*–*21*). Utilization of the FDA-approved Novavax adjuvanted subunit-based protein vaccine NVX-CoV2373 as a booster following two-dose mRNA-1273 vaccination provides a good *in vivo* test-case for mRNA-protein heterologous prime-boost. In addition, the induction of long-lived plasma cells (LLPCs) in bone marrow (BM) is vital in increasing the durability of serum antibody responses (*22*, *23*); therefore, it is crucial to develop vaccination approaches that maximize BM-LLPC production to protect against emerging SARS-CoV-2 VOCs. Non-human primate (NHP) studies are crucial in defining such vaccination strategies, as they are anatomically, physiologically, and behaviorally closer to humans.

Here, we conducted a NHP study to characterize the magnitude, breadth, and persistence of humoral and cellular immune responses induced by different booster vaccines in animals originally vaccinated with the two-dose mRNA-1273 primary series. Animals were either boosted with the homologous mRNA-1273 vaccine or adjuvanted protein-based vaccines from Novavax, NVX-CoV2373 (expressing WA-1 spike) and NVX-CoV2515 (expressing BA.1 spike). We characterized the magnitude, breadth, and durability of immune responses in the systemic and upper and lower airway mucosae collected before and after the second and third vaccination. We evaluated vaccine efficacy three months after the booster dose by challenging vaccinated and control NHP with SARS-CoV-2 BA.5 Omicron VOC. The primary goals were 1) to compare the magnitude and breadth of antibody response induced by different booster vaccinations, 2) to compare the longevity of antibody response induced by the booster with mRNA and adjuvanted NVX-CoV protein vaccines and how they influence protection against the SARS-CoV-2 BA.5 variant infection (most dominant VOC across the world at the time of the study) administered 3 months after the booster dose and 3) to determine if there is benefit of using Omicron-specific spike during booster vaccination to provide protection against the Omicron variant.

## RESULTS

### All three booster vaccines induce a strong BA.1 cross-reactive binding antibody with IgG4 dominance

**Fig. 1.**
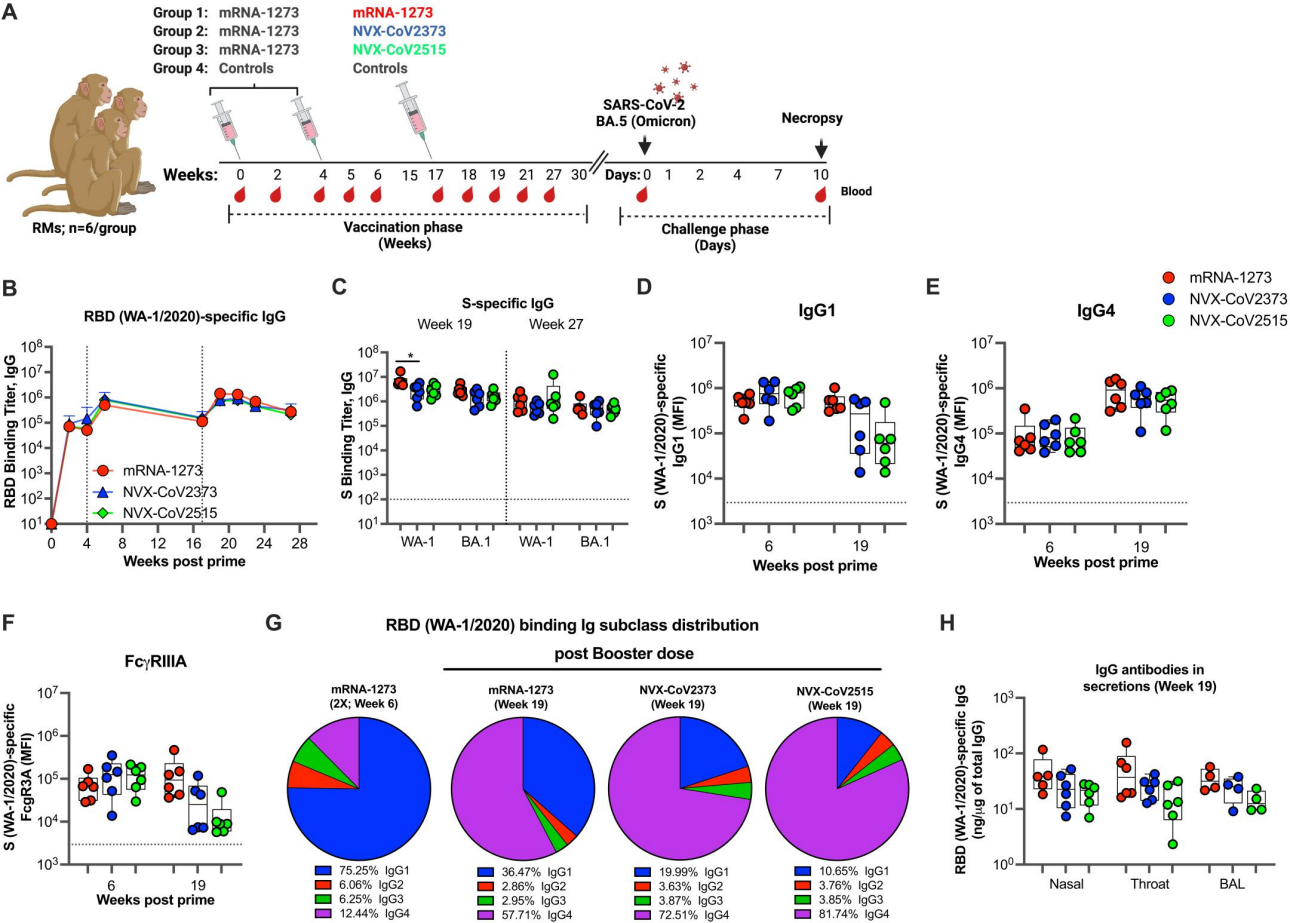
Binding antibody responses in the blood and mucosal secretions. (**A**) Schematic of the non-human primate study. Eighteen NHPs were vaccinated with mRNA-1273 at weeks 0 and 4. At week 17, animals were divided into three groups (n = 6/group). Groups 1, 2, and 3 received mRNA-1273, NVX-CoV2373, or NVX-CoV2515, respectively. An additional 6 animals were recruited at the time of challenge (Group 4), did not receive vaccination and served as the control group. At week 30, all the animals were challenged with the SARS-CoV-2 BA.5 variant via intranasal (IN) and intratracheal (IT) routes and euthanized at day 10 post-challenge. (**B**) RBD (WA-1)-specific IgG antibodies in the serum. Data are means ± SEM for each group. (**C**) WA-1 and BA.1 spike-specific binding antibody titer at weeks 19 and 27 in serum. (**D–F**) Spike-specific IgG1 (**D**), IgG4 (**E**), and FCγRIIIA binding (**F**) responses at week 19 in serum. (**G**) Distribution of IgG subclasses at weeks 6 and 19. (**H**) RBD (WA-1)-specific IgG antibody response in mucosal (nasal, throat, and lung lavage) secretions at week 19. Each dot indicates one monkey (n = 6 per group except for BAL fluids where data is available only for n = 4/group). Data represent one independent experiment. Each sample was analyzed in duplicate. Whiskers on dot plots show the maximum and minimum values. Horizontal dotted lines indicate assay limits of detection (for 2C) or geometric mean value at week 0 of the study (for 2D-2F). A two-sided Mann-Whitney rank sum test was used to compare between groups . *, p < 0.025.

Twenty-four Indian-origin male rhesus macaques (RMs), 3–5 years old, were divided into four groups (n = 6 per group) (Fig. 1A). Eighteen NHPs (groups 1–3) were administered the primary series of mRNA-1273 vaccine at weeks 0 and 4. At week 17, the group 1, 2, and 3 animals were boosted with mRNA-1273 (WA-1 matched spike, denoted in red), NVX-CoV2373 (WA-1 matched spike; denoted in blue), or NVX-CoV2515 (BA-1 matched spike; denoted in green), respectively. The NVX-CoV vaccines used in this study express full-length, prefusion stabilized, spike (S) protein trimers and are formulated with a saponin-based adjuvant, Matrix-M. The fourth group of RMs was recruited at the time of challenge, did not receive any vaccination, and served as the control group (denoted in grey). All the immunizations were performed via the intramuscular (IM) route. To measure the protective efficacy, three months after the boost, all the RMs (vaccinated and unvaccinated) were challenged with the SARS-CoV-2 BA.5 VOC. Immunological analyses for control animals prior to challenge are not available since we recruited them at the time of challenge of vaccinated animals.

We measured binding antibodies against RBD and spike proteins derived from WA-1 and BA.1 VOCs using an enzyme-linked immunosorbent assay (ELISA) at various times during vaccination. As expected and shown previously (*24*–*26*), the primary series of mRNA-1273 vaccinations induced strong RBD and spike binding antibodies at two weeks post the second dose (Wk6), and these responses contracted by about 5-fold over the next three months (Fig. 1B). All three booster vaccines induced strong RBD and spike-specific binding antibodies. At week 19 (2 weeks post-boost), the binding antibody response was boosted in all three groups. The mRNA-1273 vaccinated animals showed 2-fold higher WA-1 RBD binding antibodies compared to the NVX-CoV2515 vaccine (Fig. S1A). Similarly, the mRNA-1273 vaccinated animals had 2.5-fold higher WA-1 S binding antibodies compared to both NVX-CoV vaccines (Fig. 1C). However, at week 27, the WA-1-specific RBD and S binding responses contracted by 8 (NVX-CoV groups)- and 15 (mRNA group)-fold and were comparable between the three groups (Fig. S1A, and 1C). We next analyzed the cross-reactive binding to RBD and S proteins from BA.1 (Fig. S1A and 2C). The binding to BA.1 RBD was 3.5-, 3- and 2.6-fold lower in the mRNA-1273, NVX-CoV2373, and NVX-CoV2515 groups, respectively, compared to binding to WA-1 RBD. Similarly, binding to BA.1 S was 2.4-, 1,8-, and 1.8-fold lower in the mRNA-1273, NVX-CoV2373, and NVX-CoV2515 groups, respectively, compared to binding to WA-1 S. This resulted in relatively higher ratio of BA.1 to WA-1 S-specific antibody in both NVX-CoV groups with a statistically significant difference between NVX-CoV2515 and mRNA-1273 groups (Fig. S1B).

We next investigated the IgG subclass of RBD-binding antibodies at two weeks after the second and third (booster) doses. The primary mRNA-1273 vaccinations predominantly induced IgG1 (Fig. 1D), followed by IgG4 (Fig. 1E), with low levels of IgG2 (Fig. S1C) and IgG3 (Fig. S1D). However, the booster immunization predominantly induced an IgG4 response in all three groups. In addition, the NVX-CoV vaccines induced a 10-fold lower IgG1 response compared to the mRNA booster with a statistically significant difference between mRNA-1273 and NVX-CoV2515 groups. Consistent with a lower IgG1 response in the NVX-CoV groups, their antibodies had reduced binding to FcγRIIIA (Fig. 1F). Overall, these data demonstrate that the booster dose, regardless of vaccine, predominantly boosted the IgG4 response, and the NVX-CoV vaccines showed lower recall of IgG1 response (Fig. 1G). All three booster vaccines induced cross-reactive IgG1 and IgG4 binding antibody response against the spike and RBD proteins from other VOCs (Alpha, Beta, Delta, and Gamma and Omicron BA.1) with comparable response except that the IgG1 response in the BA.1 vaccine was significantly lower compared to the response induced by the mRNA-1273 vaccine (Fig. S2). All three booster vaccines also showed similar targeting of the binding response to N-terminal domain (NTD), RBD, S1 and S2 regions, which was predominantly directed to S1 and RBD regions (Fig. S3). We further analyzed anti-RBD binding antibodies at week 19 (2 weeks after booster) in the nasal, pharyngeal, and BAL fluids (n = 4/group) using a binding antibody multiplex assay (BAMA) and ELISA (Figs. 1H and S4). We found that all three vaccines induced comparable WA-1/2020 RBD IgG specific activity (ng antibody per μg total IgG) in mucosal secretions at the peak response (Fig. 1H).

Overall, these data demonstrate that 1) while the mRNA-1273 booster vaccine induced higher binding antibody responses than NVX-CoV protein boosters at the peak, the greater contraction in the mRNA-boosted group led to comparable titers in the memory phase (3 months post boost), 2) booster vaccination induced an IgG4 dominant response, and 3) BA.1 spike boost induced a relatively higher proportion of BA.1-specific response compared to WA-1-specific response.

### All three booster vaccines induce comparable neutralizing antibody responses against multiple VOCs including BA.5 and BQ.1.1

**Fig. 2.**
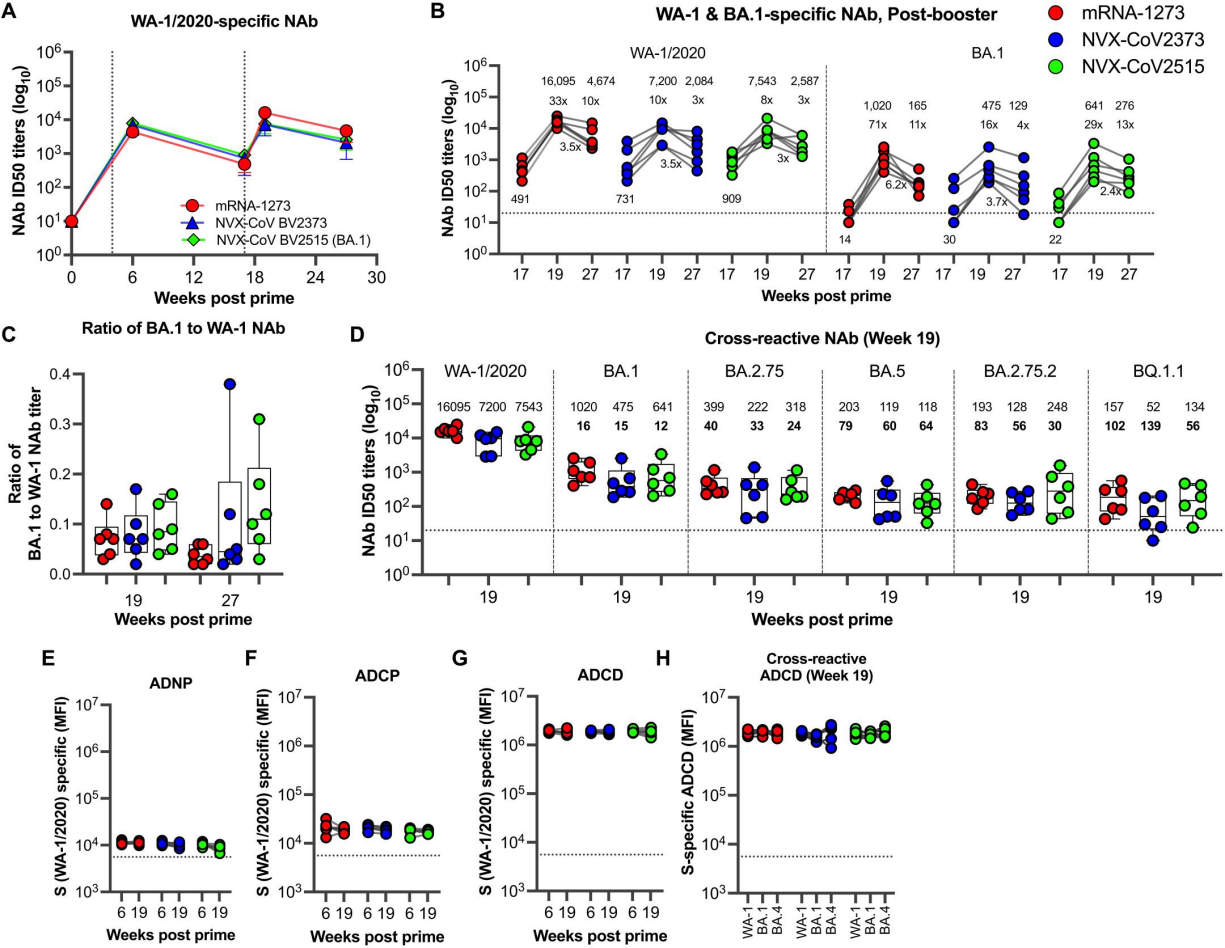
Homologous and cross-reactive functional antibody responses in the blood. (**A**) WA-1-specific live-virus neutralizing antibody (NAb) titer in serum. Data are geometric mean with error (**B**) WA-1/2020 and BA.1-specific NAb titer for individual animals at weeks 17, 19 and 27. The numbers on the top and bottom on the graph represent the geometric mean titer at each time point. The numbers with x values indicate ratios of week 19 and 27 Nab titer relative to week 17 titer. (**C**) BA.1 to WA-1 NAb titer ratios at weeks 19 and 27. (**D**) WA-1- and cross-reactive BA.1, BA.2.75, BA.5, BA.2.75.2, and BQ.1.1 VOC-specific NAb titer at week 19. The numbers on the top and bottom on the graph represent the geometric mean titer. The numbers with bold font indicate fold-change compared to WA-1. (**E – G**) WA-1 S–specific non-NAb effector functions: ADNP (**E**), ADCP (**F**), and ADCD (**G**) at weeks 6 and 19, and cross-reactive ADCD (**H**) activity at week 19. Each dot indicates one monkey (n = 6/group). Data represent one independent experiment. Each sample was analyzed in duplicate. Whiskers on dot plots show maximum and minimum values. Horizontal dotted lines indicate assay limits of detection (for 3B, 3D) or geometric mean value at week 0 of the study (for 3E-3H).

We next evaluated the neutralizing activity of sera. We performed a longitudinal analysis of WA-1/2020-specific 50% live-virus neutralization titers (ID50) to understand the neutralizing antibody (NAb) responses induced following the second dose (week 6), third dose (week 19), and pre-challenge (week 27) (Figs. 2 and S5). Consistent with previous results (*24*–*26*), the two-dose mRNA-1273 vaccination induced a robust WA-1/2020–specific NAb titer at week 6 (2 weeks after the second dose) in all vaccinated NHPs (n = 18), with a geometric mean titer (GMT) of 6,300. These responses contracted about 10-fold over three months to a titer of 680 (Fig. 2A). Consistent with the binding antibody response, all three booster vaccines induced strong WA-1/2020-specific NAb titers at week 19 (2 weeks after the third dose), with GMTs of 16,000 in mRNA-1273, 7,200 in NVX-CoV2373, and 7,500 in NVX-CoV2515 groups with the mRNA-1273 booster inducing 2.2-fold higher NAb titer compared to the NVX-CoV2373 or NVX-CoV2515 booster (Fig. 2A, 2B). This represented a 33-, 10-, 8-fold boost in the mRNA-1273, NVX-CoV2373, and NVX-CoV2515 groups, respectively at 2 weeks post boost (week 19) compared to the pre-boost time point (week 17) (Fig. 2B). These responses contracted by about 3 to 3.5-fold over three months with no significant differences between groups at the pre-challenge (week 27) timepoints (Fig. 2B). These data demonstrate that the mRNA-1273 booster dose induced marginally higher WA-1/2020-specific NAb titers compared to NVX-CoV2373 or NVX-CoV2515 boosters at the peak, but not at the memory time point (3 months post boost).

We next checked the cross-reactive NAb against BA.1 induced by all three vaccines, and whether the NVX-CoV2515 booster enhanced these responses compared to NVX-CoV2373 booster at 2 weeks post boost (week 19) (Fig. 2B). At 2 weeks post boost (week 19), 71-, 16-, and 29-fold boost in BA.1 NAb titers was observed in the mRNA-1273, NVX-CoV2373, and NVX-CoV2515 groups respectively, compared to the pre-boost time point (week 17) (Fig. 2B). The ratio of BA-1 to WA-1 NAb titer was significantly higher in the NVX-CoV2515 group compared to mRNA-1273 but not compared to NVX-CoV2373 group (Fig. 2C). The BA.1 specific NAb titer was 16-, 15- and 12-fold lower in the mRNA-1273, NVX-CoV2373 and NVX-CoV2515 groups, respectively and there was no significant difference between the three groups (Fig. 2D). These results demonstrate that BA.1 specific NVX-CoV vaccine booster did not significantly induce higher BA.1 specific NAb.

A current important goal of booster immunization is to enhance the NAb titers against VOCs. All boosters resulted in higher NAb titers to WA-1/2020 compared to VOCs, but still yielded significant cross-reactive NAb titers to BA.1, BA.5, BA.2.75, BA.2.75.2, and BQ.1.1 VOCs (Fig. 2D). Notably, the third dose induced detectable NAbs against both BA.2.75.2 and BQ.1.1, which are emerging as the predominant sub-lineages of Omicron VOCs (*1*, *2*). Irrespective of booster type, NAb titers followed the hierarchy of WA-1 > BA.1 > BA.2.75 > BA.5 > BA.2.75.2 > BQ.1.1. The respective NAb titers (GMT) to the mRNA-1273 booster were 16,095, 1,019, 399, 202, 193, and 157; to the NVX-CoV2373 booster were 7,200, 475, 221, 119, 128, and 51; and to the NVX-CoV2515 booster were 7,543, 641, 318, 118, 248, and 134, respectively (Fig. 2D). The mRNA-1273 booster induced 17-, 8- and 7.2-fold higher NAb titers to BA.1, BA.5, and BA.2.75, respectively, compared to the second dose mRNA-1273 (at week 6)-induced NAb titers (Fig. S5, C-F). Similarly, the NVX-CoV2373 booster yielded 4-, 3-, and 2-fold higher NAb titers, and the NVX-CoV2515 booster generated 4.4-, 2-, and 3.2-fold higher NAb titers to BA.1, BA.5, and BA.2.75, respectively (Fig. S5, C-F). Overall, the booster dose resulted in a greater magnitude of cross-reactive NAbs against BA.1, BA.5, BA.2.75, and BQ.1.1 isolates VOCs compared to the second dose, with no significant differences between the vaccine groups.

### All three booster vaccines induce comparable non-neutralizing antibodies with effector functions against multiple VOCs

Non-neutralizing antibody (non-NAb) Fc effector functions play an important role in protection against SARS-CoV-2 infection (*27*–*33*). We next measured the vaccine-induced non-NAb Fc effector functions at week 6 (following the two-dose mRNA-1273 vaccination) and week 19 (following a booster dose). These functions included antibody-dependent complement deposition (ADCD), antibody-dependent cellular phagocytosis (ADCP), and antibody-dependent neutrophil phagocytosis (ADNP). Notably, the two primary doses of mRNA-1273 generated low levels of ADNP (Fig. 2E) and ADCP (Fig. 2F) activities and strong ADCD activity (Fig. 2G). The booster dose induced roughly equal responses in all three groups targeting WA-1/2020 S (~1.8X10^6 MFI), VOCs BA.1 S (~1.9X10^6 MFI), and BA.4 S (~1.8X10^6 MFI) ADCD (Fig. 2H). Overall, these data indicate that the vaccination-induced antibodies exhibit durable and highly cross-reactive non-NAb Fc effector activities against SARS-CoV-2 VOCs.

### All three booster vaccines induce low frequencies of spike-specific "T cell" response in the blood

**Fig. 3.**
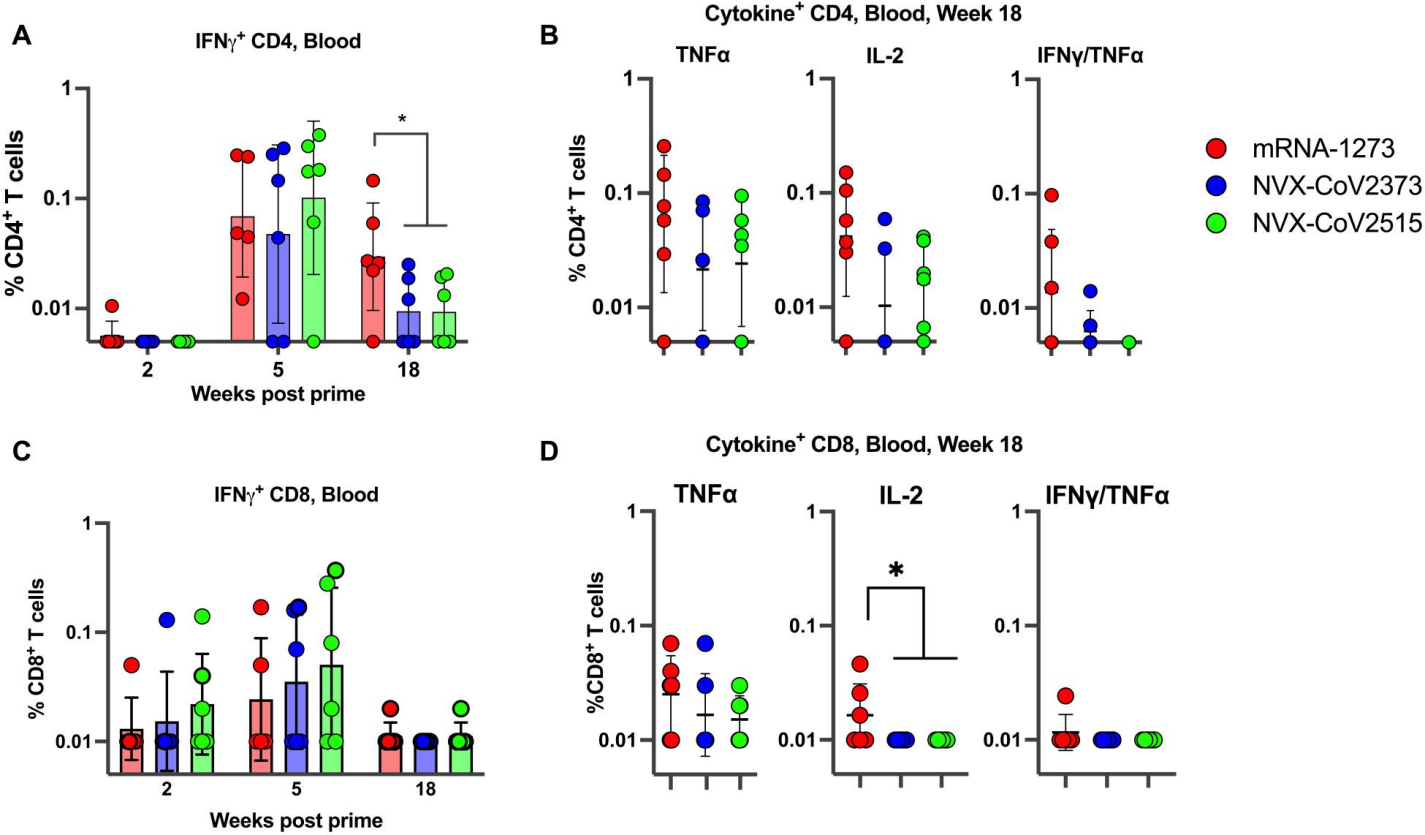
Post-vaccination antigen-specific T cell responses in blood. SARS-CoV-2 spike-specific IFNγ^+^ CD4 (**A** and **B**) and CD8 (**C** and **D**) T cell responses in the blood at weeks 2, 5, and 18 (**A** and **C**), and TNFα^+^, IL-2^+^, and IFNγ^+^/TNFα^+^ S-specific CD4 and CD8 T cell responses at week 18 (**B** and **D**). Data represent one independent experiment. Data shown are means ± SEM. A two-sided Mann-Whitney U test was used to compare groups. *, p < 0.05.

We analyzed S (WA-1/2020)-specific interferon-γ–positive (IFNγ^+^) CD4 and CD8 T cell responses in the blood (PBMCs) of vaccinated animals at weeks 2 (2 weeks after prime), 5 (1 week after the second dose), and 18 (1 week after the third dose) post-prime immunization using an intracellular cytokine staining (ICS) assay (Fig. 3). Consistent with the previous studies (*24*–*26*), our analysis showed that mRNA-1273 elicits a low frequency of S-specific IFNγ^+^ CD4 T cell response at week 5 following the two-dose primary series of vaccination with a geometric mean of 0.07 (range 0.01–0.24) (Fig. 3A). However, the magnitude of IFNγ^+^ CD4 T cell response at one week after the booster dose stayed low in all 3 groups (Fig. 3A). The group boosted with mRNA-1273 had significantly higher week 18 IFNγ^+^ responses compared to the two NVX-CoV groups (p = 0.011), indicative of a stronger Th1 response induced by the mRNA booster compared to the protein booster. S-specific interleukin-2 (IL-2) and tumor necrosis factor–α (TNF α) induction was also detected after the booster dose, but differences were not significantly different between groups (Fig. 3B). Thus, all vaccination strategies successfully induced Th1 immune responses, with a significantly stronger IFNγ^+^ CD4 T cell response in the mRNA-boosted group compared to the protein-boosted groups. The two primary series of mRNA vaccinations also induced low levels of IFNγ^+^ CD8 T cell responses in the blood and the responses were marginally higher following the second immunization compared to the first immunization (Fig. 3C). However, following the booster dose the responses were very low and mostly below our detection limit in all three groups (Fig. 3C). Similarly, the frequencies of TNFα or IL-2 producing CD8 T cells were also very low after the booster immunization (Fig. 3D). Overall, these data indicated that booster vaccination with mRNA-1273, NVX-CoV-2373, and NVX-CoV-2515 after the primary series of mRNA-1273 vaccination induces low levels of S-specific CD4 but not CD8 T cell responses.

### BA.1 vaccine induces greater proportion of BA.1 to WA-1-specific plasmablast responses compared to WA-1 vaccine

**Fig. 4.**
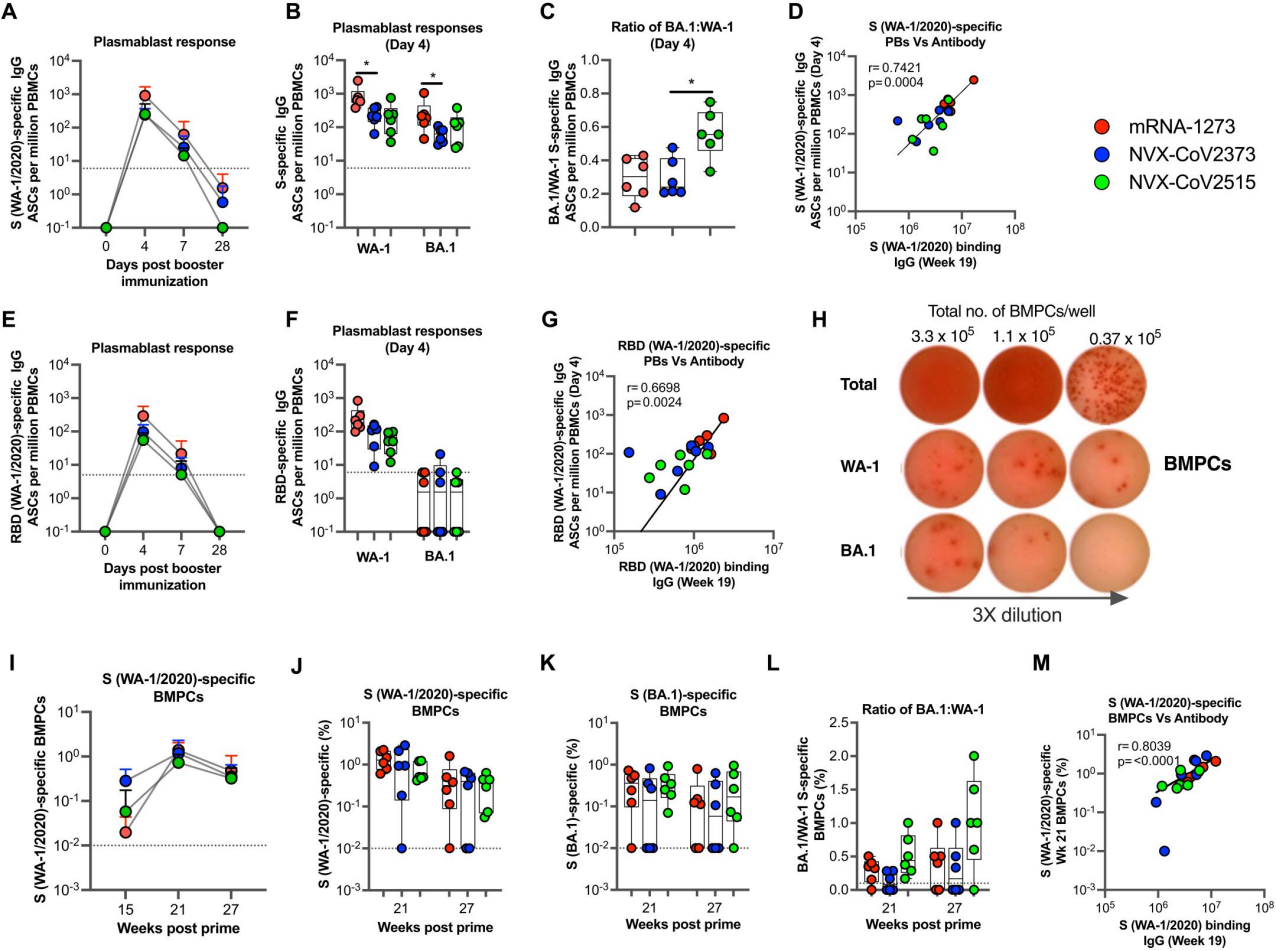
Antigen-specific antibody secreting cells (ASCs) in blood and bone marrow plasma cells (BMPCs). (**A**) WA-1 spike-specific ASCs measured by EliSpot on days 0, 4, 7, and 28 following the booster dose for each group. (**B**) WA-1 and BA.1 spike specific ASCs for individual animals at Day 4. (**C**) Ratios of BA.1 to WA-1 spike specific ASCs at Day 4. (**D**) Correlation between spike-specific binding antibody and ASCs. (**E**) WA-1 RBD-specific ASCs on days 0, 4, 7, and 28 following the booster dose for each group. (**F**) WA-1 and BA.1 RBD specific ASCs for individual animals at Day 4. (**G**) Correlation between RBD-specific binding antibody and ASCs. (**H**) Representative EliSpot images of BMPCs. (**I**) S (WA-1)-specific BMPC responses at weeks 15, 21, and 27. (**J–L**) WA-1 and BA.1 S (**J**)-specific BMPCs and their ratios (**K**) at weeks 21 and 27. (**L**) BA.1 to WA-1 S specific BMPC ratios at weeks 21 and 27. (**M**) Correlation between S (WA-1)-specific peak antibody (week 19) and BMPCs (week 21). Each dot indicates one monkey (n = 6/group). Data represent one independent experiment. Each sample was analyzed in duplicate. Whiskers on dot plots show the maximum and minimum values. Dotted lines indicate assay limits of detection. Data in A, E, and I show means ± SEM. A two-sided Mann-Whitney rank sum test was used to compare between groups . *, p < 0.025. The Spearman rank test was used for correlation analyses.

Plasmablasts (PBs) or antibody-secreting cells (ASCs) in the blood following boost represent the magnitude, specificity, and diversity of memory B cells that are being recalled (*34*, *35*). In addition, the seeding of LLPC in bone marrow (BM) is important to induce persisting serum antibody responses (*22*, *36*). We next investigated the effect of mRNA-1273, NVX-CoV2373, or NVX-CoV2515 boosting on the generation of spike-specific ASCs in the blood, and LLPCs in BM. Analysis of WA-1/2020 spike-specific IgG+ ASCs on days 0, 4, 7, and 28 following the booster vaccination showed a rapid increase on Day 4 and a sharp decline on Day 7 (Fig. 4A and S6). Consistent with the serum antibody, frequencies of WA-1/2020 S-specific ASCs were about 3.6-fold higher in the mRNA-1273 group (geomean of 742) compared to NVX-CoV2373 (geomean of 205) group at Day 4 (Fig. 4B). Further, the ASCs induced by all three vaccines showed cross-reactivity to BA.1 spike, and the ratio of BA.1 to WA-1 ASCs was 2-fold greater in the BA.1-specific vaccine compared to the WA-1-specific vaccine (Fig. 4C). The WA-1 specific ASCs at Day 4 showed a positive correlation with WA-1 spike-specific IgG in serum at 2 weeks post the booster dose (week 19) (Fig. 4D). Analysis of RBD-specific ASCs also showed similar kinetics of ASC response (Fig. 4E), higher WA-1-specific response in the mRNA-1273 group (Fig. 4F) and a positive correlation with serum WA-1 RBD-specific IgG at week 19 (Fig. 4G). As expected, all three groups showed poor cross-reactive ASCs to BA.1 RBD (Fig. 4F). These data demonstrate that the mRNA-1273 vaccine provides a stronger boost of ASCs compared to NVX-CoV vaccine. They indicate that the NVX-CoV BA.1-specific vaccine recalled a greater proportion of BA.1 to WA-1-specific memory B cells compared to NVX-CoV WA-1-specific vaccine. They also suggest that a large portion of BA.1 response was targeted outside of RBD.

Next, we evaluated LLPCs in BM on weeks 21 and 27 (four and ten weeks after the booster (Fig. 4H – 4M). The frequency of LLPCs before the boost was below our detection limit, however all three booster vaccines induced strong WA-1 spike-specific LLPCs at 4 weeks after the boost and showed a 3–6-fold decline at 10 weeks post-boost (Fig. 4I). The responses were comparable between the three groups at both time points (Fig. 4J). Like PB responses, the LLPCs also showed some cross-reactivity to BA.1 spike (Fig. 4K) and the ratio of BA.1 to WA-1 S specific LLPCs was greater in the BA.1 specific vaccine booster compared to the WA-1 specific vaccine booster (Fig. 4L). As expected, there was a strong correlation between S (WA-1/2020)-specific peak antibody at week 19 (two weeks post booster) and week 21 LLPCs (four weeks post booster) (Fig. 4M). Overall, these analyses showed that all booster vaccines induced strong LLPCs in BM and suggested that BA.1 specific vaccine induces a relatively higher proportion of BA.1 specific LLPCs.

### All three booster vaccines protect against SARS-CoV-2 BA.5 infection in the lower airway

To determine the extent of protection provided by different booster vaccines, we challenged vaccinated and control NHPs at week 30 (3 months after the booster) with SARS-CoV-2 BA.5 via intranasal (IN) and intratracheal (IT) inoculation. BA.5 was the dominant VOC in mid-2022, with a spike sequence more closely related to BA.1 than to WA-1/2020 and with higher transmissibility and neutralization resistance compared to prior VOCs. Successive variant evolution has resulted in differential disease severity between different VOCs in animal models (*37*), and it is unknown whether BA.5 infects and causes pathogenesis in nonhuman primates (NHPs).

**Fig. 5.**
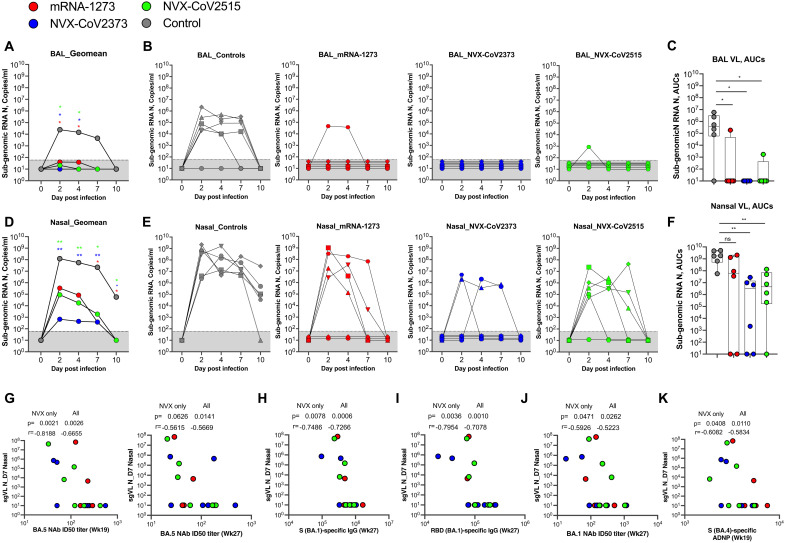
Efficacy of booster vaccines against BA.5 infection in respiratory airways. Subgenomic nucleocapsid RNA (sgRNA N) of SARS-CoV-2 BA.5 VOC viral loads (sgRNA N copies/ml) in BAL (**A–C**) and nasal swabs (**D–F**) post-challenge. Viral loads are shown as geomean for each group (**A** and **D**), for individual animals (**B** and **E**), and as the area under the curve (AUC) (**C** and **F**). The AUC was calculated using viral load from day 0 to day 10. Each dot indicates one monkey (n = 6/group except on Day 10 post-infection (necropsy) nasal swabs, where data is available for five animals in mRNA-1273 (n = 5/group) and four animals in NVX-CoV2373 (n = 4/group) group. Data represent one independent experiment. Each sample was analyzed in duplicate. Whiskers on dot plots show the maximum and minimum values. Dotted lines in A, B, D, and E indicate assay limits of detection. (**G–K**) Correlation between Day 7 viral load in nasal swabs and week 19 and week 27 BA.5 NAb titer (**G**), S (BA.1)-specific IgG (**H**), RBD (BA.1)- specific IgG (**I**), week 27 BA.1 NAb titers (**J**) and week 19 BA.4 spike-specific ADNP activity (**K**). A two-sided Mann-Whitney rank sum test was used to compare between control and respective vaccinated groups. ns, not significant; *, p < 0.05; **, p < 0.01. P values were not corrected for multiple comparisons since we did not make comparisons between two vaccine groups. The Spearman rank test was used for correlation analyses.

We measured nucleocapsid-specific subgenomic RNA (sgRNA N) in the nasopharynx (upper airway) and bronchoalveolar lavage (BAL) (lower airway) on days 0, 2, 4, 7, and 10 (day of euthanasia) post-infection, and compared the viral loads in the upper and lower airway between vaccinated and control animals (Fig. 5). We observed that the virus replication peaked at day 2 in both the BAL (Fig. 5B) and nasopharynx (Fig. 5E) of unvaccinated control NHPs, with the viral loads reaching a geometric mean copy number of 2.4x10^4^ (Fig. 5A) and 1.2x10^8^ (Fig. 5D) sgRNA N copies per mL, respectively. Five of six (5/6) in the lower airway and six of six (6/6) control NHPs in the upper airway showed virus replication. All animals had detectable viremia in the upper airway at all times tested until necropsy, along with 5/6 animals in the lower airway, indicating productive BA.5 VOC infection in NHPs. After the challenge, the mRNA-1273, NVX-CoV2373, and NVX-CoV2515 boosted animals showed markedly lower virus replication compared to controls in the lower (Fig. 5A, 5B, and 5C) and the upper (Fig. 5D, 5E, and 5F) airways, starting from day 2, with a significant difference on days 2 and 4 in the lower airways and until necropsy in the upper airways. Strikingly, in lower airways (BAL), all vaccinated animals showed profound viral control, with just one of six animals from the mRNA-1273 and NVX-CoV2515 groups having any detectable virus (Fig. 5B). Remarkably, none of the NVX-CoV2373 vaccinated animals had detectable viral loads in the lower airways (Fig. 5B).

### NVX-CoV2373 and NVX-CoV2515 vaccines blunt viral replication in the upper airway

As has been observed with previous vaccines, the viral control was variable in the upper airways (*24*–*26*), yet, three doses of vaccination lowered both the peak viremia and improved viral control at day seven compared with the unvaccinated NHPs (Fig. 5D, 5E, and 5F). Notably, the heterologous vector-based booster vaccination with NVX-CoV2373 or NVX-CoV2515 resulted in significantly lower viral loads in the upper airways starting from day 2 and until necropsy, compared to unvaccinated animals (Fig. 5D, 5E, and 5F). Surprisingly, a majority (four of six) of the mRNA-1273-boosted animals had variable but high viral loads (2.6x10^6^ to 1x10^9^ sgRNA N copies per mL) in the upper airways (Fig. 5E). Further, the NVX-CoV2373 booster provided superior protection in the upper airways, with virus detected only in three animals (Fig. 5E).

Additionally, the area under the curve (AUC) of viral load in BAL and nasopharynx between days 0 to 10 was markedly lower in all the vaccinated animals compared to controls (Fig. 5C and 5F). Similarly, enhanced protection from BA.5 infection was observed in the nasopharynx (upper respiratory airways) of NVX-CoV2373 and NVX-CoV2515 vaccinated animals compared to control animals (Fig. 5F). All the animals were euthanized on day 10 following the SARS-CoV-2 BA.5 challenge, and evaluated for lung pathology and viral antigen as described previously (Fig. S7, A – D; Table S1) (*15*, *24*, *38*). Four of the six control animals showed mild to moderate signs of inflammation, and two showed moderate to severe inflammation. Three out of 6 controls also showed rare to occasional foci of viral antigen. In contrast, all of the NVX-CoV-vaccinated RMs, irrespective of the type of booster vaccine received, showed mild to moderate lung pathology, and 3 out of 12 animals showed rare to occasional foci of viral antigen. However, 3 out of 6 RMs from the mRNA-1273 booster group showed moderate to severe lung pathology and rare to occasional foci of viral antigen. In addition, body weights and respiratory rates remained stable after the challenge (Fig. S8). Together, these data indicated that the booster vaccination with either mRNA-1273 or NVX-CoVs provides protection from virus replication in the airways.

### Immune correlates for protection

Post-challenge at euthanasia, the binding (Fig. S9A) and neutralizing antibody (Fig. S9B) responses were comparable between the three groups, and these responses were mostly below our detection limit in the unvaccinated controls. However, the control animals showed induction of spike-specific IFNγ, TNFα, and IL-2-producing CD4 T cells in the blood, BAL, lung, and hilar LNs, and these responses were generally higher compared to vaccinated animals (Fig. S10). The post-challenge antibody and "T cell" responses are consistent with profound viral control/protection in the vaccinated animals.

We performed correlations between various immune measurements post-vaccination and viral control in the lower and upper airways (Fig. 5, G – K). We found some important associations with the sgRNA N loads in the nasal compartment and vaccine-induced antibody responses weeks 19 (2 weeks post booster dose) and 27 (pre-challenge). The BA.5 specific neutralizing activity at week 19 and week 27 correlated inversely with Day 7 viral loads (Fig. 5G). The binding IgG specific to BA-1 spike (Fig. 5H) and BA-1 RBD (Fig. 5I) at week 27, and BA.1 specific neutralizing activity at week 27 (Fig. 5J) and BA.4 Spike-specific ADNP activity at week 19 (Fig. 5K) also correlated inversely with Day 7 viral loads. We found additional correlations between WA-1 specific binding activity, neutralization titer against WA-1 and BA.2.75, and Day 7 viral loads (Fig. S11). However, we did not find any significant associations with viral load in the lower airway since nearly all vaccinated animals showed viral loads below the detection level (Fig. S12). These data suggested that multiple antibody functions, including binding, neutralizing, and ADNP activities, contributed to enhanced protection against SARS-CoV-2 BA.5 infection in the upper airway.

Overall, these results demonstrate that a booster with either the mRNA-1273, NVX-CoV2373, or NVX-CoV2515 vaccine protects from virus replication in the lower airway even after 3 months post-vaccination, and a booster with both NVX-CoV2373 and NVX-CoV2515 vaccines provides superior viral control in the upper airway against BA.5 VOC infection in NHPs.

## DISCUSSION

To the best of our knowledge, this is the first NHP study directly comparing an mRNA-1273 booster to an adjuvanted protein-based vaccine booster in animals previously vaccinated with mRNA-1273. We designed this study to understand the magnitude, breadth, and durability of the antibody response induced by these two delivery platforms and their influence on the upper and lower airway protection against the heterologous BA.5 challenge administered 3 months after the booster. In addition, we designed the study to address whether a BA.1 spike-matched booster might perform better than the original strain against BA.5 challenge. Importantly, given that most human infections occur during periods of waning immunity, we elected to challenge animals three months after their final booster dose to better assess the maintenance of protection. This is in contrast to the majority of SARS-CoV-2 NHP studies conducted so far, which challenged animals during the peak of vaccine-induced antibody response at four weeks after the boost. Our results showed that all three booster vaccines enhance cross-neutralizing activity against multiple VOCs, including BA.5 and BQ 1.1 (the most dominant VOCs at the current time), and provide strong protection in the lower airway. They also showed that Novavax vaccines blunt viral replication in the upper airway. Overall, these results highlighted the near-term (3 months) protective ability of mRNA-1273 and NVX-CoV2373 booster doses against BA.5 challenge.

A key question for COVID-19 vaccination strategy is to address whether there is a need to change the vaccine to match the spike in the circulating VOCs. Our results using the BA.1 variant-specific spike vaccine booster did not show significant benefit in enhancing the cross-neutralization against the BA variants and protection against BA.5. Our observations align well with recent reports where booster vaccination with mRNA-1273 or a VOC-matched vaccine both induce comparable neutralizing antibody responses against the VOC and protect the lower airway against beta (B.1.351) or Omicron (BA.1: B.1.1.529) challenges (*15*, *25*). The BA.1 Omicron variant contains 32 spike protein mutations, most located in neutralizing antibody epitopes (*39*, *40*). Because of this, we expect that memory B cells that are common to both WA-1 and BA.1 spikes will be preferentially boosted. This does not translate into higher neutralizing antibody titer against BA.1 since the majority of serum antibody is derived from bone marrow plasma cells that were predominantly specific to WA-1 spike. However, our plasmablast analysis at Day 4 post boost showed that the BA.1-specific booster recruited a relatively higher proportion of BA.1-specific memory B cells than WA-1-specific memory B cells. These results strongly suggest that a second booster dose with a BA.1-specific vaccine could potentially induce a higher magnitude of cross-reactive neutralizing antibody responses against BA variants, compared to an ancestral spike-specific boost.

One of the goals of our study was to compare the magnitude, breadth, and longevity of the immune response induced between the booster vaccines. Previous studies (*15*, *16*, *25*, *41*) showed that three doses of mRNA-1273 significantly improved the WA-1/2020-specific neutralization titers compared to two-doses, which we also observed here. In addition, all three booster vaccines enhanced the cross-reactive neutralizing antibody titers against VOCs, including BA.1, BA.2.75, BA.2.75.2, BA.5, and BQ.1.1, with detectable neutralization titers three months after the booster dose and with no significant differences between booster vaccines. While the mRNA-1273 booster showed a stronger neutralizing antibody response against the ancestral WA-1 spike compared to the NVX vaccines, this was not true for neutralizing antibody responses against other VOCs, highlighting some differences between the two platforms. Interestingly, there were no differences in the magnitudes of non-neutralizing antibody responses such as ADCD, ADCC, and ADCP following two and three doses of vaccination, indicating that these responses were not as significantly boosted as the neutralizing antibody response. This could be due to a change in the dominance of IgG subclass of the antibody from IgG1 to IgG4 following the boost. In general, we found that more vaccine doses appeared to be linked to the induction of higher spike-associated IgG4 antibody responses, irrespective of vaccine booster vaccine. Interestingly, recent studies in humans also showed similar IgG4 dominance following booster immunizations with BNT162b2 and mRNA-1273 mRNA vaccinations (*42*–*44*). These results highlight similarities between human and NHP studies in modulating IgG class switching during vaccination. It was interesting to note that the booster vaccine type did not significantly alter the specificity of antibody response with respect to the major regions of spike with the majority of the antibody response targeting the RBD region. Booster type did not alter binding to RBD region in spite of the presence of many mutations within the Omicron RBD in NVX-CoV2515. Overall, the effect of the third dose was to improve neutralizing antibody titers against VOCs and increase IgG4-specific anti-spike antibodies, all without substantially changing domains being targeted on spike protein. Higher-resolution methods, such as electron microscopy–based polyclonal epitope mapping (EMPEM) may be needed to better characterize differences in antibody specificities between different vaccination regimens.

While neutralizing antibody titers appear to be severely affected by mutations in the Omicron VOCs, studies have shown that T cell epitopes are more conserved, suggesting that T cell immunity plays a role in limiting severe disease in the absence of neutralizing antibody epitopes (*45*–*47*). Consistently, our study showed that all three booster vaccinations induced similar CD4 T cell responses. We observed a bias towards Th1 cells, which may be necessary for the control of SARS-CoV-2 VOCs. Notably, the IFNγ+ CD4 T cell responses were significantly higher in mRNA-1273 boosted animals than the combined NVX-CoV boosted animals (NVX-CoV2373 and NVX-CoV2515), suggestive of a stronger Th1 response when boosting with mRNA.

One of the important findings of our study is that NVX vaccines blunt virus replication in the upper respiratory airway early post-infection. Importantly, all three vaccines showed a profound control of virus replication in the upper respiratory airway by 7–10 days, while the control animals showed high levels of persisting viral loads. Consistent with previous studies (*24*–*26*), the mRNA-1273 booster offered only limited protection early post-infection (at Days 2 and 4) in the upper airways. However, both NVX vaccines showed significant control of viral load in the upper airways starting from Day 2 until necropsy. Nasopharyngeal viral loads correlate with the presence and quantity of infectious viruses (*48*); thus, vaccines that reduce viral loads early during infection are likely to help reduce transmission to other individuals. Our results highlight the need to study NVX vaccines’ impact on viral load in the upper respiratory airway in a larger cohort of NHPs and humans. The mechanisms that contributed to enhanced viral control in the upper respiratory airway of NVX vaccinated animals are not completely clear. One possibility is that the NVX vaccines had higher levels of spike-specific IgG in the nasal secretions at the time of challenge.

In summary, our data demonstrate that a booster dose with either mRNA-1273 or NVX-CoV (2373 or 2515), following a primary series of mRNA-1273, elicits broadly cross-reactive and durable humoral immune responses and protects NHPs against SARS-CoV-2 BA.5 VOC infection. Notably, our results suggest that the NVX-CoV2373 (WA-1 matched) booster provides greater protection in the airways, with superior viral control in the upper respiratory airways compared to controls. These data support the use of all currently available boosters for the prevention of infection and transmission of disease from circulating and emerging SARS-CoV-2 VOCs, and suggest that adjuvanted protein boosters may be a preferred option to maximize protection.

## MATERIALS AND METHODS

### Study design

We designed this study to evaluate the immunogenicity of mRNA-1273, NVX-CoV2373 (WA-1 spike matched), or NVX-CoV2515 (BA.1 spike matched) booster vaccination and their efficacy against SARS-CoV-2 BA.5 variant challenge in NHPs. To mimic a significant fraction of SARS-CoV-2 vaccinated individuals in the US, we vaccinated animals initially with two-doses of mRNA-1273 to establish a primary series of vaccination before the booster dose was administered. We challenged animals with SARS-CoV-2 BA.5 VOC since it was the dominant VOC across the world at the time of challenge. We characterized the magnitude and breadth of cellular and humoral immune responses following vaccination and challenge, to define immune correlates for protection against heterologous SARS-CoV-2 BA.5 infection. We used six animals per group on the basis of our previous experience in detecting immune responses and assessing protective efficacy in vaccine studies in macaques. We collected various samples, including blood, mucosal swabs (nasal and throat), bronchoalveolar lavage (BAL) fluid, and bone marrow multiple times post-vaccination and challenge. In general, these included collections on the day of and 1–2 weeks after each vaccination and at euthanasia (Day 10 post-challenge). Following the challenge, we collected samples from the nasopharynx and BAL every 2–3 days until euthanasia to measure the viral loads in the upper and lower respiratory tract, respectively. We collected blood, BAL, and lung tissue at necropsy to evaluate post-challenge cellular and humoral immune responses. In addition, we assessed lung pathology and the SARS-CoV-2 antigens at euthanasia using histology on the day and necropsy.

### EXPERIMENTAL DESIGN

#### Rhesus macaque (RM) model and immunizations

Twenty-four Indian-origin male rhesus macaques (*Macaca mulatta*), 3–5 years old, were evenly divided into four groups of six animals each, matched on age and weight. Groups 1–3 were administered the primary series of two doses of 100 μg of mRNA-1273 (WA-1-matched spike) at weeks 0 (prime) and 4 (boost). At week 17, groups 1, 2, and 3 were boosted with 50 μg of mRNA-1273 (WA-1 matched spike), 5 μg NVX-CoV2373 (WA-1 matched spike), and 5 μg NVX-CoV2515 (BA-1 matched spike), respectively. Groups 2 and 3 also received 50 μg Matrix-M adjuvant as part of their protein vaccines. The fourth group of RMs were recruited at the time of challenge, received no vaccine and served as the control group. NVX-CoV2373 and NVX-CoV2515 were co-formulated with Matrix-M adjuvant and single-dose vials were shipped at 2-8C. All immunizations were performed via the intramuscular (IM) route, and a conventional 25-gauge needle (0.5- or 1-ml volume) was used to deliver the vaccines.

#### SARS-CoV-2 BA.5 challenge in RMs

The twenty-four RMs (vaccinated and unvaccinated) were reorganized into five viral challenge cohorts. Each cohort contained a mix of unvaccinated and vaccinated animals, except cohorts 1 (n = 3, all controls) and 2 (n = 3, all vaccinated) contained 3 RMs per group. Cohorts 3, 4, and 5 had 6 RMs per group, which included one control and five vaccinated animals. All animals were housed at the Emory National Primate Research Center (ENPRC) of Emory University under BSL2 conditions during the vaccination phase and were moved to BSL-3 suite at least one week before the challenge for acclimatization. The time interval between the final vaccination and challenge was 13 weeks, 5 days for all animals except for the three vaccinated animals in challenge cohort 2, where the interval was between 12 to 14 weeks.

At week 30, 13 weeks after the final immunization, the macaques were challenged with a total of 6x10^5 PFUs of SARS-CoV-2 BA.5 variant (Omicron BA.5 VOC, titered on Vero-TMPRSS2 cells). The virus was administered as 2 ml by the intratracheal (IT) route and 1 ml by the intranasal (IN) route (0.5 ml in each nostril). Nasopharyngeal swabs and BAL samples were collected, stored immediately in an RNA/DNA shield, and processed for viral RNA extraction. After the viral challenge on day 0, nasopharyngeal swabs and BAL fluid were collected in an RNA/DNA shield on days 2, 4, 7, and 10, and their viral loads were measured. On Day 10, after the SARS-CoV-2 challenge, all vaccinated and non-vaccinated macaques were euthanized. Necropsy samples (lung tissues) were collected and stained with Hematoxylin and Eosin.

### Animal subjects

All animals were living in standard NHP cages and were provided with both standard primate feed (Jumbo Monkey Diet 5037; Purina Mills, St. Louis, MO), fresh fruit, and enrichment daily, as well as free access to water. Trained research and veterinary staff performed immunizations and blood draws, with other sample collections performed under anesthesia with ketamine (5 to 10 mg/kg) or telazol (3 to 5 mg/kg).

### Ethics Statements

Animal experiments were approved by the Emory University Institutional Animal Care and Use Committee (IACUC). Emory National Primate Research Center is an AAALAC-accredited facility. All animal experiments were carried out by USDA regulations and recommendations derived from the Guide for the Care and Use of Laboratory Animals.

### Cells and Viruses

Vero-TMPRSS2 cells were cultured in complete DMEM medium consisting of 1x DMEM (VWR, #45000–304), 10% FBS, 2 mM L-glutamine, and 1x antibiotic as previously described (*49*, *50*). nCoV/USA_WA1/2020 (WA/1), closely resembling the original Wuhan strain, was propagated from an infectious SARS-CoV-2 clone as previously described (*51*). icSARS-CoV-2 was passed once to generate a working stock. The BA.1 isolate has been previously described (*52*). Omicron subvariants were isolated from residual nasal swabs: BA.5 isolate (EPI_ISL_13512579), provided by Dr. Richard Webby (St Jude Children’s Research Hospital), BA.2.75.2 (EPI_ISL_15146622), BQ.1.1 isolate (EPI_ISL_15196219), and BA.2.75 isolate (EPI_ISL_14393635) provided by Dr. Benjamin Pinsky (Stanford University). All variants were plaque purified and propagated once in VeroE6-TMPRSS2 cells to generate working stocks. Viruses were deep sequenced and confirmed as previously described (*53*).

#### Enzyme-linked immunosorbent assay (ELISA) for serum antibodies

SARS-CoV-2 S and RBD–specific IgG in serum were quantified by enzyme-linked immunosorbent assay (ELISA) as described previously (*28*). Briefly, Nunc high-binding ELISA plates were coated with 2 μg/ml of recombinant SARS-CoV-2 (RBD and S) proteins in Dulbecco’s phosphate-buffered saline (DPBS) and incubated overnight at four °C. Plates were then blocked with 5% blotting-grade milk powder and 4% whey powder in DPBS with 0.05% Tween 20 for two hours at room temperature (RT). Plates were then incubated with serially diluted serum samples (starting from 100, 3-fold, 8x) and incubated for two hours at RT, followed by six washes. Total SARS-CoV-2 S (RBD and S)-specific monkey IgG antibodies were detected using HRP-conjugated anti-monkey IgG secondary antibodies (1:10,000), respectively, incubated for one hour at room temperature (RT). The plates were washed and developed for 30 minutes using TMB (2-Component Microwell Peroxidase Substrate Kit), and the reaction was stopped using 1 N phosphoric acid solution. Plates were read at 450 nm wavelength within 30 min using a plate reader (Molecular Devices, San Jose, CA, USA). ELISA endpoint titers were the highest reciprocal serum dilution that yielded an absorbance >2-fold over background values.

### Binding antibody multiplex assay (BAMA) and ELISA for mucosal antibodies

A customized BAMA was used to measure IgG antibodies specific for the RBD of SARS-CoV-2 WA-1/2020 and VOCs BA.1 and BA.5 variants in secretions. Each protein was coupled to Bio-Plex Pro magnetic carboxylated beads containing a particular ratio of fluorescent dyes and incubated overnight with diluted samples and standard as described (*54*). The standard was calibrated using anti-RBD humanized IgG monoclonal antibodies and consisted of pooled IgG purified from macaques previously immunized with COVID vaccines. Beads were developed using biotinylated goat anti-human IgG followed by neutralite avidin-phycoerythrin (both SouthernBiotech) as described (*54*). Fluorescence was recorded, and standard curves were constructed using a Bioplex 200 (Bio-Rad). Antibody concentrations were calculated and subsequently normalized relative to the total IgG concentration in the sample. Total IgG was measured by ELISA as described by (*55*) using goat anti-monkey IgG (AlphaDiagnostics) to coat plates, rhesus IgG (Rockland) as standard, and the above biotinylated antibody, avidin-peroxidase and TMB (SouthernBiotech) to develop plates.

#### Live-virus neutralization

FRNT assays were performed as previously described (*56*). Briefly, samples were diluted at 3-fold in 8 serial dilutions using DMEM in duplicates with an initial dilution of 1:10 in a total volume of 60 μl. Serially diluted samples were incubated with an equal volume of SARS-CoV-2 (100–200 foci per well) at 37^o^ C for 1 hour in a round-bottomed 96-well culture plate. The antibody-virus mixture was then added to Vero cells and incubated at 37^o^ C for 1 hour. Post-incubation, the antibody-virus mixture was removed and 100 μl of prewarmed 0.85% methylcellulose overlay was added to each well. Plates were incubated at 37^o^ C for 18 to 40 hours, and the methylcellulose overlay was removed and washed six times with PBS. Cells were fixed with 2% paraformaldehyde in PBS for 30 minutes. Following fixation, plates were washed twice with PBS, and permeabilization buffer (0.1% BSA, 0.1% Saponin in PBS) was added to permeabilize cells for at least 20 minutes. Cells were incubated with an anti-SARS-CoV spike primary antibody directly conjugated to Alexa Fluor-647 (CR3022-AF647) overnight at 4°C. Cells were then washed twice with 1x PBS and imaged on an ELISPOT reader (CTL Analyzer). Antibody neutralization was quantified by counting the number of foci for each sample using the Viridot program (*57*). The neutralization titers were calculated as follows: 1 – (ratio of the mean number of foci in the presence of sera and foci at the highest dilution of the respective sera sample). Each specimen was tested in duplicate. The FRNT-50 titers were interpolated using a 4-parameter nonlinear regression in GraphPad Prism 9.2.0. Samples that do not neutralize at the limit of detection at 50% are plotted at 20 and used for geometric mean and fold-change calculations.

#### Luminex assay to quantify the antigen-specific antibody isotype, IgG subclass, IgA, IgM, and FcγR

A Luminex assay was used to detect and quantify antigen-specific subclass, isotype, and Fc-receptor (binding) factors (*58*). Carboxylate-modified microspheres (Luminex) were carboxy-coupled to the different SARS-CoV-2 antigens. Immune complexes were formed by mixing appropriately diluted plasma (1:100 for IgG1, IgG2, IgG3, IgG4, IgA, IgM, and 1:1000 for FcγRs) to antigen-coupled beads and incubating the complexes overnight at 4°C. Immune complexes were then washed in PBS with 0.1% BSA and 0.02% Tween-20. Murine secondary antibodies for each antibody isotype or subclass were used to detect antigen-specific antibody titer and probed with a PE-conjugated tertiary anti-mouse antibody (Invitrogen). For FcγRs, biotinylated FcγRs were labeled with streptavidin-PE before addition to immune complexes. Fluorescence was measured with an iQue (Intellicyt) and analyzed using Forecyt software. Data are reported as median fluorescence intensity (MFI).

#### ADCP, ADNP, and ADCD Assays for monkey sera

Antibody-dependent cellular phagocytosis (ADCP), antibody-dependent neutrophil phagocytosis (ADNP), and antibody-dependent complement deposition (ADCD) were measured as previously described by (*59*–*61*). Neutravidin beads were coupled to biotinylated SARS-CoV-2 antigens. Antigen-coupled beads were then incubated with appropriately diluted plasma (ADCP 1:200, ADNP 1:50, ADCD 1:10) for 2 hours at 37°C to form immune complexes. For ADCP, 2.5x10^4^ THP-1 s cells were added and incubated for 16 hours at 37°C. For ADNP, leukocytes were isolated from fresh peripheral whole blood by lysing erythrocytes using ammonium-chloride potassium lysis. Leukocytes were added to immune complexes at 5x10^4^ cells/well and incubated for 1 hour at 37°C. Neutrophils were detected using anti-human CD66b Pacific-Blue. For ADCD, lyophilized guinea pig complement was resuspended, diluted in gelatin veronal buffer with calcium and magnesium (GVB++, Boston BioProducts), and added to immune complexes. The deposition of C3 was detected using an anti-C3 FITC antibody.

All functional assays were acquired with an iQue (Intellicyt) and analyzed using Forecyt software. For ADCP, events were gated on singlets and fluorescent cells. For ADNP, bead-positive neutrophils were defined as CD66b positive fluorescent cells. For both ADCP and ADNP, a phagocytic score was expressed as (percentage of bead-positive cells) x (MFI of bead-positive cells) divided by 10000. For ADCD, data were reported as median fluorescence of C3 deposition (MFI).

### Intracellular Cytokine Staining (ICS) assay

Functional responses of SARS-CoV-2 S1 and S2-specific CD8^+^ and CD4^+^ T cells in vaccinated animals were measured using peptide pools and an intracellular cytokine staining (ICS) assay. Please refer to the Supplementary Materials for detailed methods.

### Enzyme-linked immunospot (ELISpot) assay

ELISPOT assays were performed as previously described (*62*) with few modifications. Please refer to the Supplementary Materials for detailed methods.

### Viral RNA extraction and quantification

SARS-CoV-2 subgenomic RNA (sgmRNA) was quantified in nasopharyngeal (NP) swabs, throat swabs, and broncho-alveolar lavages (BAL). Please refer to the Supplementary Materials for detailed methods.

### Histopathology and immunohistochemistry

Histopathology and detection of SARS-CoV-2 virus antigen were performed as previously described (*15*, *24*, *38*). Briefly, lung tissue sections were processed and stained with hematoxylin and eosin (H&E) for pathological analysis and with a rabbit polyclonal anti-SARS-CoV-2 anti-nucleocapsid antibody in immunohistochemistry (IHC) staining the presence of virus antigen. The polyclonal antibody (GeneTex, GTX135357) was used at a dilution of 1:2000. The tissue sections used for gross histology examination include the left cranial lobe (Lc), right middle lobe (Rmid), and right caudal lobe (Rc). The extent and severity of alveolar inflammation were characterized by the criteria the number of lung lobes affected, type 2 pneumocyte hyperplasia, alveolar septal thickening, fibrosis, perivascular cuffing, peribronchiolar hyperplasia, inflammatory infiltrates, and hyaline membrane formation. Each lung lobe was assessed individually for animals with multiple affected lung lobes, and then the scores were by presence and absence of signs for inflammation and virus antigen. Tissue sections were analyzed by a blinded board-certified veterinary pathologist using an Olympus BX43 light microscope. Photomicrographs were taken on an Olympus DP27 camera.

### Quantification and statistical analysis

Throughout the manuscript, we compared mRNA-1273 and NVX-CoV2373 groups to determine differences between the two platforms, and NVX-CoV2373 and NVX-CoV2515 groups to determine differences between the two spike proteins. Accordingly, we used a threshold P value of less than 0.025 as being significant to correct for multiple comparisons. The difference between any two groups at a time point was measured either using a two-tailed nonparametric Mann–Whitney rank-sum test. Comparisons between different time points within a group used paired parametric t-tests. A p-value of less than 0.05 was considered significant for comparisons between different time points within a group. The sample n is listed in corresponding figure legends. The correlation analysis was performed using the Spearman rank test. GraphPad Prism version 8.4.3 (GraphPad Software) was used for data analysis and statistics.

## Supplementary Materials

### Other Supplementary Material for this manuscript includes the following:

Data file S1

MDAR Reproducibility Checklist

## Notes

View/request a protocol for this paper from *Bio-protocol*.
