# Supplementary material for: Efficacy of mRNA-1273 and Novavax ancestral or BA.1 spike booster vaccines against SARS-CoV-2 BA.5 infection in non-human primates
Source: Sci Immunol. 2023 May 16:eadg7015. doi: 10.1126/sciimmunol.adg7015 (PMC10451060; doi:10.1126/sciimmunol.adg7015)
Supplement: Supplementary file 1 — Materials and Methods Figs. S1 to S12 Table S1 [file sciimmunol.adg7015_sm.pdf]

Science Immunology
AAAS

Supplementary Materials for

Efficacy of mRNA-1273 and Novavax ancestral or BA.1 spike booster vaccines against SARS-CoV-2 BA.5 infection in non-human primates

Nanda Kishore Routhu *et al.*

Corresponding author: Rama Rao Amara, ramara@emory.edu

Sci. Immunol. , eadg7015 (2023)
DOI: 10.1126/sciimmunol.adg7015

The PDF file includes:

Materials and Methods
Figs. S1 to S12
Table 1

Other Supplementary Material for this manuscript includes the following:

Data file S1
MDAR Reproducibility Checklist

Supplementary information

Materials and Methods

Samples processing

PBMCs from blood collected in sodium citrate CPT tubes for macaques were isolated using standard procedures. Post-SARS-CoV-2 challenge, samples were processed and stained in a BSL-3 facility. To collect and process BAL fluids for single-cell isolation, up to 50 ml of physiological saline was delivered through the trachea to the lungs of anesthetized animals using a camera-enabled fiberoptic bronchoscope. The flushed saline was re-aspirated five times before pulling out the bronchoscope. Lungs were cut into small pieces and incubated at 37 °C in RPMI (1×) medium containing Collagenase type IV and DNase I with gentle shaking for 30 min. After incubation, cells were isolated by forcing tissue suspensions through a 70 μM cell strainer. RBCs were removed by ACK lysis buffer, and live cells were counted by trypan blue exclusion. For processing lymph nodes, lymph-node biopsies were dissociated by passing through a 70μm cell strainer. All the cell suspensions were filtered through a 70μm cell strainer and centrifuged at 2200 rpm for 5 minutes. Pelleted cells were suspended in 1ml R10 medium (RPMI (1X), 10% FBS) and stained as described in individual sections.

Intracellular Cytokine Staining (ICS) assay

Functional responses of SARS-CoV-2 S1 and S2-specific $CD8^+$ and $CD4^+$ T cells in vaccinated animals were measured using peptide pools and an intracellular cytokine staining (ICS) assay. Overlapping peptides from spike protein (13 or 17-mers overlapping by ten amino acids) were obtained from BEI resources (NR-52402), and different pools (S1 and S2) were made. The S1 pool contained peptides mixed from 1-97, and the S2 pool combined from 98-181 peptides. The stimulation reaction used each peptide at a 1 mg/ml concentration. Two million cells suspended in 0.2 ml of RPMI 1640 medium with 10% FBS were stimulated with 1 μg/ml CD28, 1 μg/ml CD49d co-stimulatory antibodies, and different peptide pools. These stimulated cells were incubated at 37°C in a 5% CO2 incubator. After 2 hrs of incubation, 1 μl Golgi-plug and 1 μl Golgi-stop/ml were added, and samples were incubated for 4 more hours. After 6 hours of incubation, cells were transferred to 4°C overnight and stained the next day. Cells were washed once with FACS wash (1X PBS, 2% FBS, and 0.05% sodium azide) and surface stained with Live/Dead-APC-Cy7 (ThermoFisher L34976), anti-CD4-BV650 (custom conjugate), and anti-CD8-BUV496 (BD-Horizon 612942), each conjugated to a different fluorochrome for 20 minutes at RT. The stained cells were washed once with FACS wash and permeabilized with 0.2 ml of Cytofix/Cytoperm for 20 minutes at 4°C. Cells were washed once with perm wash and incubated with anti-cytokine (IFNγ-A700 custom conjugate, TNFα-PE-TR custom conjugate, IL-2-PeCy7 (Biolegend 500326), IL-4-PE (Miltenyi Biotec 130-123-698), IL-17 FITC (eBiosciences 11-7179-342), IL-21 Alexa-Fluor 647 (BD 560493), and CD40L-BV605 (Biolegend 310826)) and anti-CD3-PerCP-Cy5.5 (BD 552852) antibodies for 20 minutes at 4°C. Finally, the samples were washed once with perm wash and once with FACS wash and fixed in 4% paraformaldehyde for 20 minutes before acquisition on a BD LSR Fortessa flow cytometer. Data were analyzed using FlowJo software. Total signal was reported by subtracting the background signal NS (from non-stimulated cells) from the signal of the stimulated cells (S1 or S2). S1-NS and S2-NS had to be greater than NS in order to be reported as positive signal and the difference had to represent at least 5 positive cells. Total S signal was calculated by combining (S1-NS) + (S2-NS) and also had to exceed NS to be reported as positive. Otherwise, samples were reported at the detection limit of the assay.

Enzyme-linked immunospot (ELISpot) assay

ELISPOT assays were performed as previously described (*63*) with few modifications. The SARS-CoV-2 WA-1/2020 S (40589-V08H4, Sino Biological) and RBD (40592-V08H, Sino Biological)

and B.1.1.529 S (NR-56447 BEI Resources), and RBD (Lab purified) proteins were diluted to 1 mg/ml, and total Ig (LS-347340 LifeSpan Bioscineces Inc) was diluted to 5 mg/ml in sterile 1X phosphate buffered saline (PBS). The ELISpot plates (MSHAN4B50, Millipore-Sigma) were coated with 100 ml/well and incubated for 2 hours. The plates were then washed with wash buffer I (1X PBS, 0.05% Tween-20) one time and three times with wash buffer II (1X PBS). The plates were then blocked with 100 ml per well of complete RPMI (10% FBS, 1X Pen/Strep in 1X RPMI) and incubated in a 5% CO2 incubator at 37°C for two h. The peripheral blood mononuclear (PBMCs) or bone marrow (BM) cells were resuspended at 10^7 cells/ml. Then, 50ml (total of ~5x10^5 cells/well) of cell suspension per well was added to the first well of each column with successive 3-fold dilutions thereafter. After 16 to 18 h incubation, the plates were washed four times with each wash buffer I and II. The plates were incubated with a biotin-conjugated anti-monkey IgG antibody (in 1:2000, Cat#617-106-012, Rockland) for two h at room temperature (RT). After washing the plates four times with wash buffer I containing 1% fetal calf serum (FCS), the plates were incubated with horseradish peroxidase (HRP) Avidin D (in 1:5000, Cat#A-2004, Vector Laboratories) for two hours at room temperature (RT). The secondary antibody and the HRP Avidin D were diluted using wash buffer I containing 1% fetal calf serum (FCS). Then, the plates were washed four times with wash buffer I and developed using AEC (0.3 mg/ml AEC in 0.1M Na-Acetate buffer, pH 5.0, and 0.03% Hydrogen Peroxide). The plates were dried and detected using the Immunospot CTL counter and Image Acquisition 4.5 software (Cellular Technology). Spots were counted manually. ASCs were reported as per million PBMCs or percentage of total bone marrow plasma cells.

Viral RNA extraction and quantification

SARS-CoV-2 subgenomic RNA (sgmRNA) was quantified in nasopharyngeal (NP) swabs, throat swabs, and broncho-alveolar lavages (BAL). In brief, following swab collection, the sample was placed in 200uL 1X DNA/RNA Shield (Zymo Cat#R1100). For broncho-alveolar lavage (BAL), 2X DNA/RNA Shield (Zymo Cat#R1200) was added at equal volume to the sample (200uL BAL Sup: 200uL 2X DNA/RNA Shield). Samples were heat-inactivated before processing at 65ºC for 60 minutes per Institutional Biosafety stipulations. RNA was then extracted from the samples using the Zymo Viral RNA Kit (Zymo #R1035) according to the manufacturer's instructions. Viral RNA Buffer (Zymo Catg#R1034) was added at twice the volume of the original sample (200uL sample - 400uL buffer). BAL samples were then incubated for 30min before being added to Zymo IC spin column (Zymo Cat# C1004). Swab samples were processed without incubation. The spin column was washed twice with Wash Buffer (Zymo Cat# R1003) before ethanol addition and elution in 50uL DNase/RNase-free water. Samples were stored at -80ºC until plating and viral load quantification.

Isolated RNA was analyzed in a QuantStudio 6 RT PCR system (Thermo Scientific, USA) using 5uL of sample plated with 1.8uL forward primer, 1.8 reverse primers (Integrated DNA Technologies), 0.5uL probe, 5uL TaqPath 1-Step RT-qPCR (Fisher Cat#A15299), and 5.9uL ddH2O for a total 20uL reaction with the following program: 25°C for 2 minutes, 50°C for 15 minutes, 95°C for 2 minutes followed by 40 cycles of 95°C for 3 seconds and 60°C for 30 seconds. Signals were compared to a standard curve generated using in vitro transcribed RNA of each sequence diluted from 10^8 to 10 copies. Positive controls consisted of SARS-CoV-2 infected VeroE6 cell lysate. Viral copies per swab were calculated by multiplying mean copies per well by the amount in the total swab extract. The following primers and probes were used in the assay to quantify the subgenomic RNA N copies: sgm-N FOR 5'-CGATCTCTTGTAGATCTGTTCTC-3'; sgm-N PRB 5′-/56-FAM-CGATCAAAACAACGTCGGCCCC-3BHQ-1/-3′ ; sgm-N REV 5'-GGTGAACCAAGACGCAGTAT-3'.

Supplementary figures

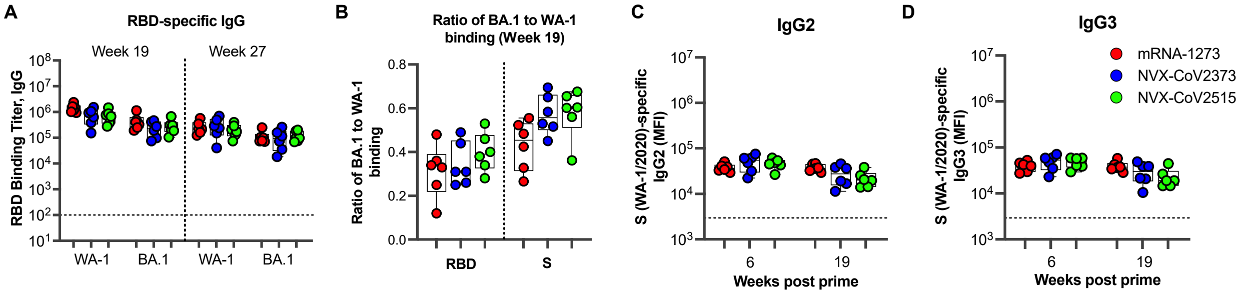

Figure S1. Booster vaccine-induced binding antibody responses in the blood. (**A**) WA-1 and BA.1 RBD specific binding antibody titer following booster dose at weeks 19 and 27 in serum. (**B**) Ratio of BA.1 to WA-1 binding titer at week 19. (**C** and **D**) Spike-specific IgG1 (**C**), and IgG3 (**D**) responses at week 19 in serum. Each dot indicates one monkey. Data represent one independent experiment. Each sample was analyzed in duplicate. Whiskers on dot plots show the maximum and minimum values. Horizontal dotted lines in S1A indicate assay limits of detection, or in S1C and S1D, indicate assay limits geometric mean value at week 0 of the study.

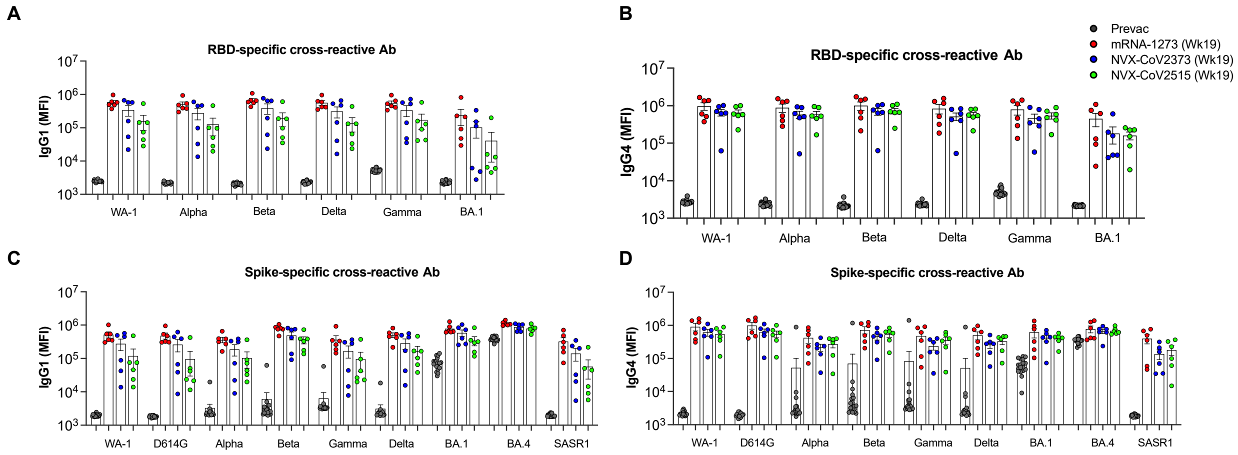

Figure S2. S and RBD of WA-1/2020 and VOC binding antibody responses. (**A–D**) RBD and S of WA-1/2020 and VOCs binding IgG1 (**A** and **C**), and IgG4 (**B** and **D**) subclass antibodies in the sera at weeks 0 and 19. Each dot indicates one monkey (n=18 at week 0; n=6/group at week 19). Data represent one independent experiment. Each sample was analyzed in duplicate.

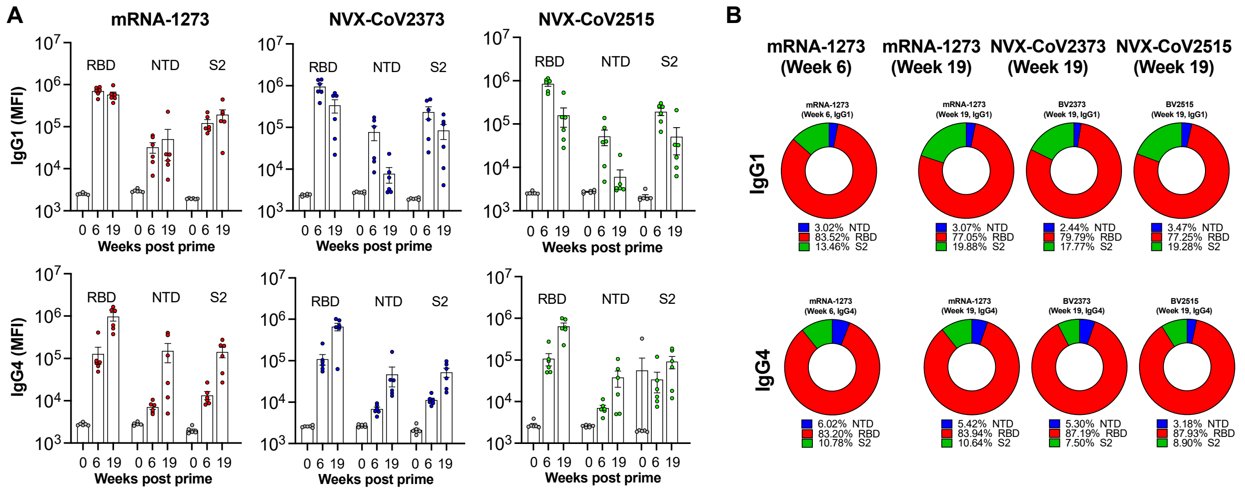

Figure S3. Specificity of S (WA-1/2020)-specific antibodies. (**A**) Scatter plots showing the IgG1 and IgG4 binding antibody against three domains/regions of S (WA-1/2020) protein in the vaccinated animals at weeks 0, 6 and 19. (**B**) Pie graphs showing the proportions of IgG1 and IgG4 binding antibodies for the data shown in A. The domains include the N-terminal domain (NTD), receptor binding domain (RBD), and S2 regions. Each dot in **A** indicate one monkey, 6 RMs per group. Data represent one independent experiment. Each sample was analyzed in duplicate.

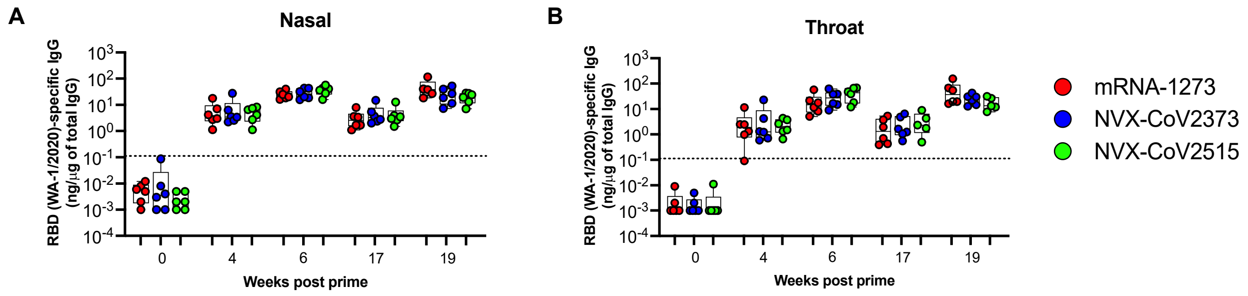

Figure S4. IgG antibodies in mucosal secretions. (**A–B**) Longitudinal analysis of RBD (WA-1/2020)-specific IgG in nasal (**A**) and throat (**B**) swabs expressed as a ratio of total IgG. Each dot indicates one monkey (n=6/group). Data represent one independent experiment. Each sample was analyzed in duplicate. Whiskers on dot plots show the maximum and minimum values. Horizontal dotted lines (geometric mean value) indicate assay limits, as it was calculated using pre-vaccination (Week 0) serum.

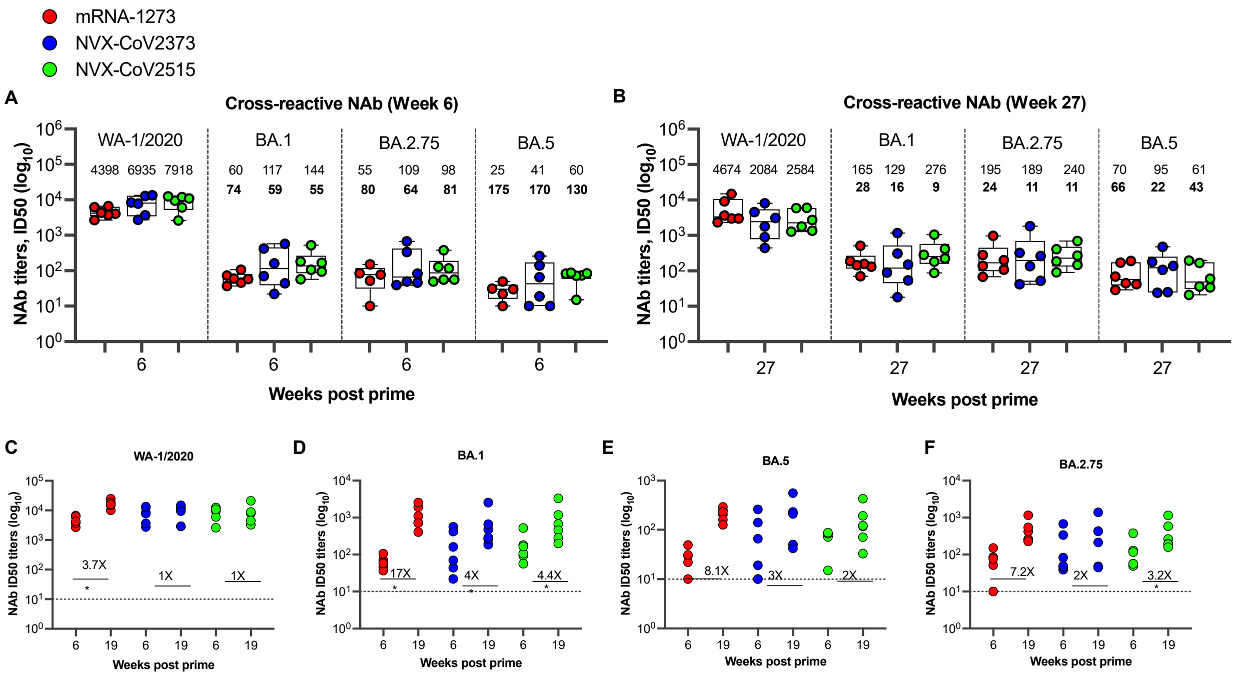

Figure S5. WA-1/2020 and Omicron VOCs cross-reactive neutralizing antibody responses. (**A**, **B**) WA-1/2020 and VOC-reactive NAb responses in sera at weeks 6 (**A**) and 27 (**B**), with mean NAb titer values labeled above and fold-change (compared to WA-1/2020) underneath in bold. Each dot indicates one monkey (n=6/group). Data represent one independent experiment. Each sample was analyzed in duplicate. Whisker plots show maximum and minimum values. **(C-F)** WA-1/2020 and Cross-reactive BA.1, BA.5, and BA.2.75–specific cross-neutralization activity between weeks 6 and 19. A two-sided Mann-Whitney U test was used to compare time points. **, $p < 0.01$, ns =not significant. The fold-differences are indicated between the timepoints. Horizontal dotted lines indicate assay limits of detection.

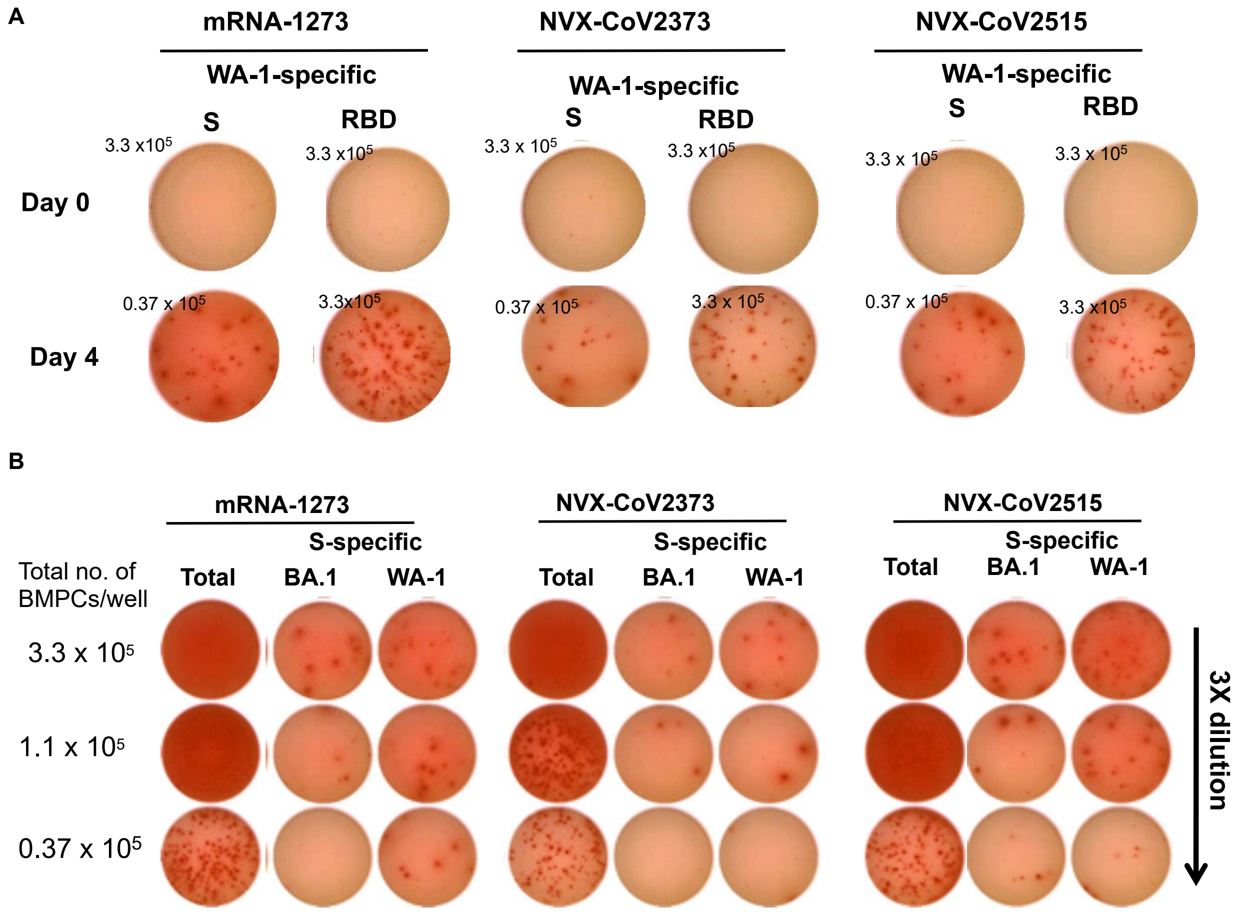

Figure S6. Representative ELISpot images. (**A**) Plasmablast responses in the blood, and **(B)** plasma cell responses in the bone marrow aspirates.

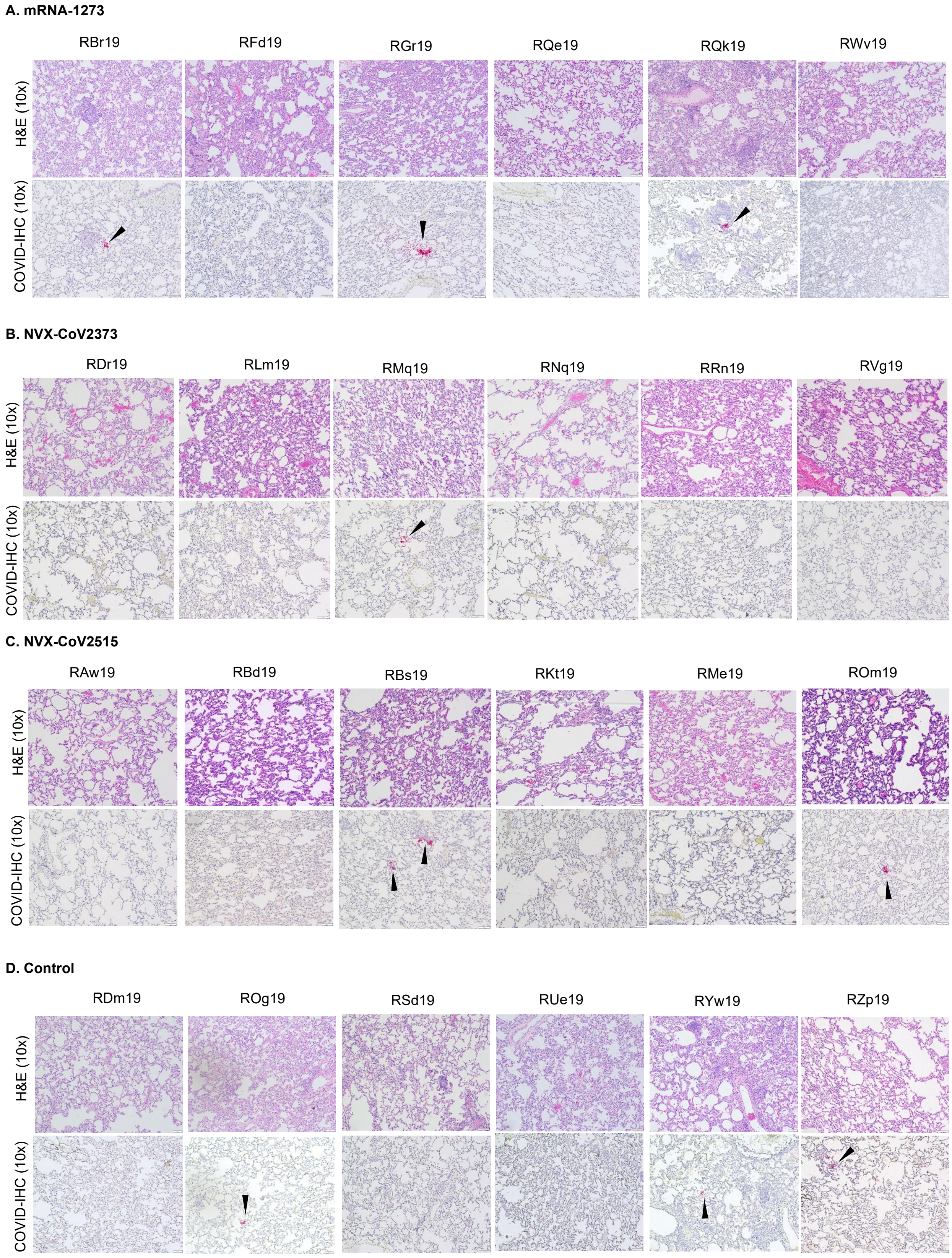
A. mRNA-1273
RBr19
RFd19
RGr19
RQe19
RQk19
RWv19
H&E (10x)
COVID-IHC (10x)
B. NVX-CoV2373
RDr19
RLm19
RMq19
RNq19
RRn19
RVg19
H&E (10x)
COVID-IHC (10x)
C. NVX-CoV2515
RAw19
RBd19
RBs19
RKt19
RMe19
ROm19
H&E (10x)
COVID-IHC (10x)
D. Control
RDm19
ROg19
RSd19
RUe19
RYw19
RZp19
H&E (10x)
COVID-IHC (10x)

Figure S7. Evaluation of lung pathology and presence of viral antigen in the lung. Lung sections were taken from (**A**) mRNA-1273, (**B**) NVX-CoV2373, (**C**) NVX-CoV2515 vaccinated or (**D**) control animals (n=6 per group) following euthanasia on day 10 post-challenge and were used to analyze tissue structure and cell infiltration using hematoxylin and eosin stain (H&E) and the presence of virus antigen by immunohistochemistry (IHC). Upper: H&E stain illustrating the extent of the inflammation and cellular infiltrates. Images at 10× magnification with black bars for scale (100 µm). Lower: representative images indicating SARS-CoV-2 N antigen detection by IHC with a polyclonal anti-N antibody. Antigen-positive foci are indicated by an arrow.

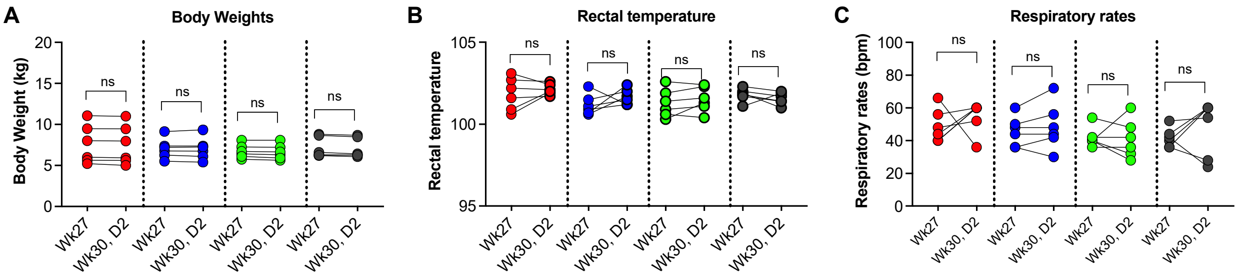

Figure S8. Body weights and temperatures of RMs before and after SARS-CoV-2 BA.5 challenge. Body weight **(A),** body temperatures **(B)**, and respiratory rates **(C)** of RMs (control and vaccinated) were collected before and after the challenge. Each dot indicates one monkey (n=6 per group. Data represent one independent experiment. A two-sided Mann-Whitney U test was used to compare between groups. ns=non-significant.

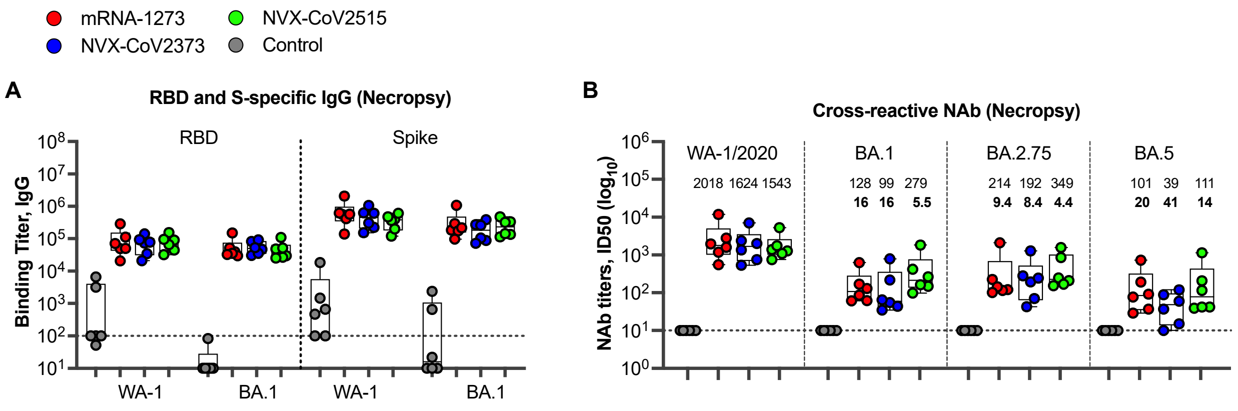

Figure S9. Post-challenge WA-1/2020 and Omicron VOCs cross-reactive binding and neutralizing antibody responses post BA.5 challenge. (**A**) RBD and S of WA-1/2020 and BA.1-reactive binding antibody responses, (**B**) WA-1/2020 and VOC-reactive NAb titers, at day 10 after the BA.5 challenge. The mean NAb titer values are shown above and fold-change (compared to WA-1/2020) values are shown below these values in bold. Each dot indicates one monkey (n=6/group). Data represent one independent experiment. Each sample was analyzed in duplicate. Whiskers on dot plots show maximum and minimum values. Horizontal dotted lines indicate assay limits of detection.

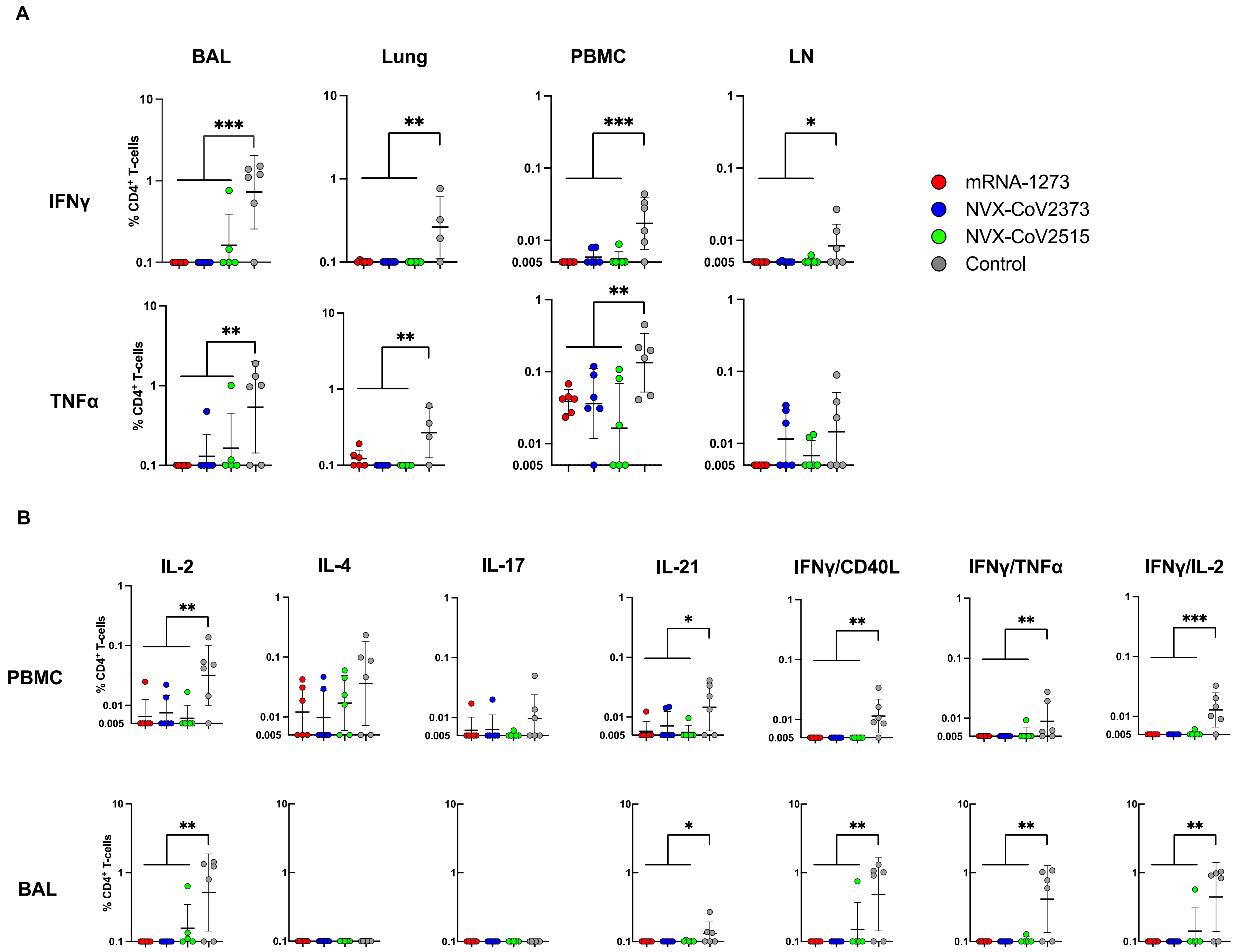

Figure S10. Post-challenge spike-specific CD4 T cell responses in various tissue compartments. (A) SARS-CoV-2 S–specific IFNγ+ (upper) and TNFα+ (lower) CD4 T cell responses in the bronchoalveolar lavage (BAL), Lung tissue, blood, and hilar lymph node of vaccinated and control animals at day 10 post-infection (necropsy). (**B**) SARS-CoV-2 S–specific IL2, IL4, IL-17, IL-21, IFNγ, and IL21 cytokine-positive CD4 T cell responses, as well as the double-positive responses of IFNγ/CD40L, IFNγ/ TNFα, and IFNγ/IL-2 in the blood (upper) and BAL (lower) of vaccinated and control animals at day 10 post-infection (necropsy). Data are means ± SEM and represent one independent experiment. A two-sided Mann-Whitney rank sum test was used to compare groups. *, $p<0.05$; **, $p<0.01$; and ***, $p<0.001$

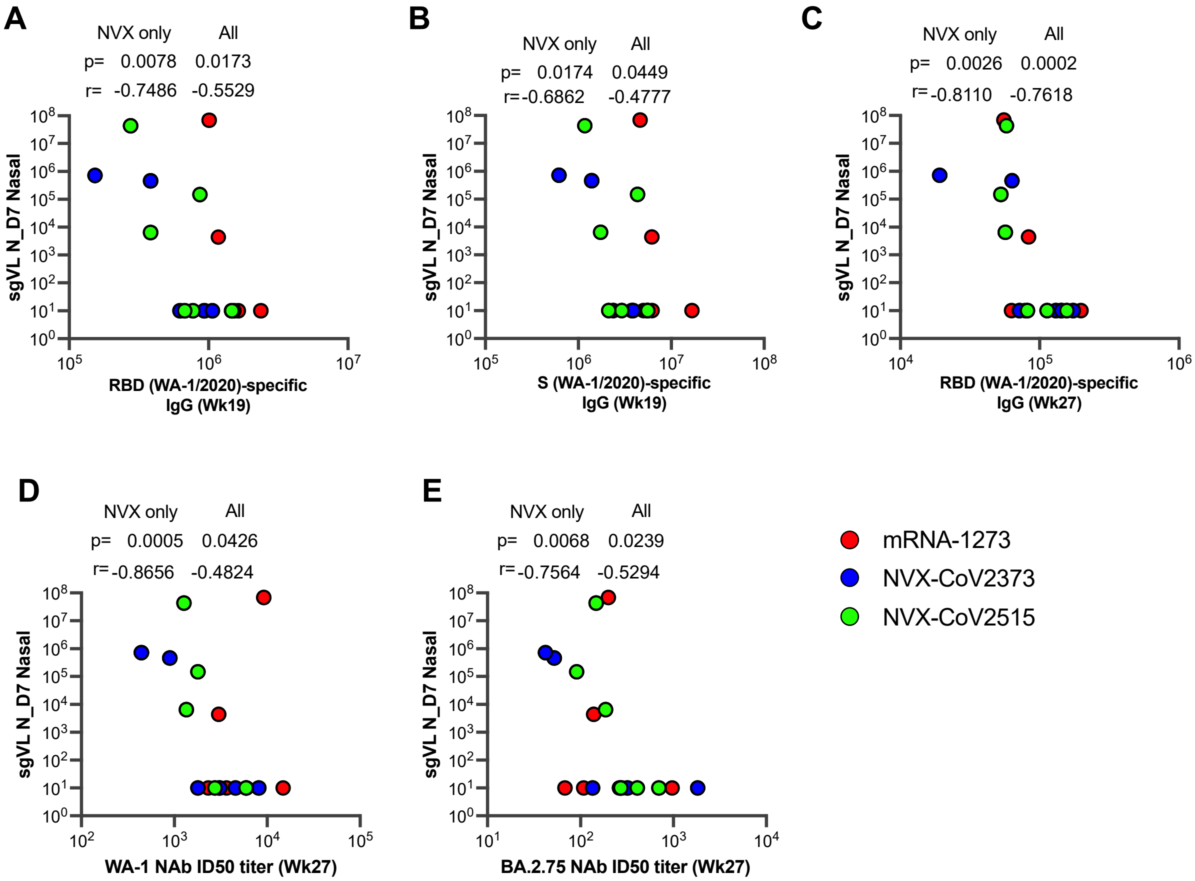

Figure S11. Correlation analysis between antibody immune responses and viral loads in nasal secretions. Circles represent individual NHPs. The Spearman rank test was used for correlation analyses. r is Spearman's correlation coefficient and p is the corresponding *p*-value.

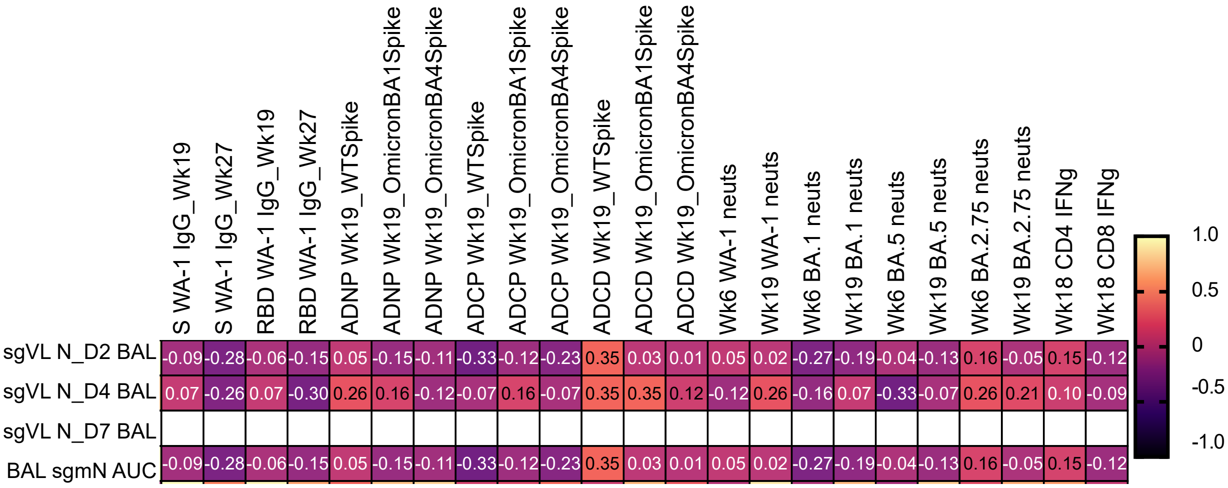

Figure S12. Correlation matrix analysis between sgRNA N loads in BAL fluids and antibody and T cell responses. The color refers to the r value scale (−1 to 1) shown on the right. The number in each cell indicates the actual r value. White open boxes indicate correlations where r values could not be determined.

Supplementary tables

Group	Animal ID	H&E scores			IHC (N antigen) scores			sgRNA N AUCs	
		Lc	Rmid	Rc	Lc	Rmid	Rc	BAL	Nasal
mRNA-1273	RBr19	++	+	++	-	+/-	-	10	35,058,268
	RFd19	+	+	+/-	-	-	-	10	89,333,423
	RGr19	++	+	++	+/-	-	+/-	10	10
	RQe19	+/-	+/-	+/-	-	-	-	10	10
	RQk19	+	++	+++	+/-	+/-	+/-	187,332	1,315,506,674
	RWv19	+	+	+	-	-	-	10	2,070,211,308
NVX-CoV2373	RDr19	+/-	+/-	+/-	-	-	-	10	2,175
	RLm19	+	+	+	-	-	-	10	9,799,606
	RMq19	+	+	+	+/-	-	-	10	10
	RNq19	+/-	+/-	+/-	-	-	-	10	27,722,841
	RRn19	+	+	+/-	-	-	-	10	6,481,368
	RVg19	+	+	+/-	-	-	-	10	10
NVX-CoV2515	RAw19	+/-	+	+/-	-	-	-	10	10
	RBd19	+	+	+/-	-	-	-	10	7,524,562
	RBs19	+	+	+	-	-	+/-	1,728	220,502
	RKt19	+/-	+/-	+/-	-	-	-	10	1,363,933
	RMe19	+	+	+	-	-	-	10	48,989,231
	ROm19	+	+	+	+/-	-	-	10	133,678,414
Control	RDm19	+	+/-	+	-	-	-	76,708	654,941,649
	ROg19	+	+	++	+/-	+/-	+/-	2,377,557	1,691,414,529
	RSd19	+	+	+	-	-	-	525,357	4,634,680,390
	RUe19	+	+/-	+	-	-	-	10	1,811,237,569
	RYw19	++	+	++	+/-	-	+/-	5,841,816	5,055,034,920
	RZp19	+/-	+/-	+	-	-	+/-	227,871	56,080,937

Note:
Left cranial lobe (Lc) of the lung;
Right middle lobe (Rmid) of the lung;
Right caudal lobe (Rc) of the lung

Scoring	H&E (Inflammation)	IHC (N antigen)
-	Minimal to absent	No foci
+/-	Minimal to mild	Rare to occasional foci
+	Mild to moderate	Foci
++	Moderate to severe	Occasional to multiple
+++	Severe	Multiple to numerous

Table 1. Lung pathology scores at necropsy. The lung inflammation and virus antigen scores were assessed from lung tissue collected following necropsy (Day 10 after challenge), includes the left cranial lobe (Lc), right middle lobe (Rmid), and right caudal lobe (Rc). Inflammation (H&E staining) scoring legend: − absent to minimal inflammation; +/− minimal to mild inflammation; + mild to moderate inflammation; ++ moderate-to-severe inflammation; +++ severe inflammation. Antigen (IHC staining) scoring legend: − no antigen detected; +/− rare to occasional foci; + occasional to multiple foci; ++ multiple to numerous foci; +++ numerous foci. Horizontal rows correspond to individual rhesus macaques from that particular treatment group.
